# Early applications of apple vision pro in clinical medicine and medical education: a scoping review

**DOI:** 10.3389/fmed.2026.1909038

**Published:** 2026-10-16

**Authors:** Yiwei Hu, Yaqin Wang, Xiaohua Zuo, Haibo Hu, Bo Min, Chengxiang Wang, Zilong Tang, Ning Xiao, Chenjun Huang, Chuxu Wang

**Affiliations:** 1 Department of Thoracic Surgery, The First Affiliated Hospital with Nanjing Medical University, Nanjing, China; 2 Department of Thoracic Surgery, Huai’an Second People's Hospital, The Affiliated Huai’an Hospital of Xuzhou Medical University, Huai’an, China; 3 Department of Pain Management, Huai’an Second People's Hospital, The Affiliated Huai’an Hospital of Xuzhou Medical University, Huai’an, China; 4 The First School of Clinical Medicine, Nanjing Medical University, Nanjing, China

**Keywords:** apple vision pro, clinical medicine, medical education, mixed reality, precision medicine

## Abstract

The Apple Vision Pro (AVP), a next-generation spatial computing device, has demonstrated potential applications in multidisciplinary clinical diagnosis, treatment, and medical education, by leveraging its unique technical architecture and performance advantages. In clinical practice, its fundamental advantages of three-dimensional (3D) visualization, real-time image integration, and immersive interaction have been preliminarily employed in disease diagnosis and treatment, surgical planning, precise operations, and postoperative rehabilitation across multiple disciplines. In medical education, AVP surpasses the limitations of conventional models, offering a novel methodology for anatomy education, clinical skills development, specialized practical training, and collaboration of distance learning, thereby alleviating persistent challenges such as unequal distribution of teaching resources, low efficiency in skills and specialized training, and the high cost of distance education. However, AVP faces some limitations, including limited hardware portability, insufficient system compatibility, a lack of specialized medical applications, high costs, and incomplete medical certification. Currently, most evidence derives from feasibility studies, case reports, and opinion pieces. Only two Stage 2b confirmatory studies were identified. Thus, high-level evidence for clinical benefit remains scarce. Readers should interpret all reported benefits as preliminary technical demonstrations rather than established clinical outcomes. Future advancements necessitate iterative development of lightweight hardware design, development of specialized medical software, cross-system optimization, and establishment of standardization protocols. Moreover, more randomized controlled trials (RCTs) and multicenter studies are urgently needed to fully explore and validate its intrinsic value in precision medicine and the equitable allocation of medical resources.

## 1 Introduction

In clinical medicine, conventional diagnostic and therapeutic approaches are sometimes restricted by limitations such as inadequate vision of anatomical structures, reliance on two-dimensional (2D) imaging for surgical planning, and unsatisfactory immersive experiences in remote guidance (1, 2). In medical education, significant challenges include a lack of cadaver resources, hazards in clinical skills training, and unequal distribution of resources across regions (3, 4). As digital health technologies rapidly advance, extended reality (XR) technologies are progressively surpassing traditional medical models, providing novel solutions to these challenges (5, 6).

The concept of XR, proposed by Steve Mann and Charles Wyckoff in 1991, covers virtual reality (VR), augmented reality (AR), and mixed reality (MR). VR is a fully immersive 3D virtual environment characterized by immersion, interactivity, and imagination. AR overlays virtual objects onto the real world without interaction. MR, situated between VR and AR, integrates both features, enabling deep integration and natural interaction between real and virtual objects in the same space (7).

Based on the above definitions, the AVP is a representative MR head-mounted device and has quickly become a research focus in medicine due to its integration of AR and VR. To date, AVP has been applied in disease diagnosis, surgical planning, precise manipulation, and postoperative rehabilitation within multiple clinical specialties. Its application has progressively expanded to medical education.

This study systematically reviews the research progress of AVP applications in clinical medicine and medical education, evaluates its technical advantages and existing limitations, and identifies gaps in current evidence. It aims to provide references for clinical translation, technical optimization and large-scale deployment of the device, and underscores the necessity of conducting high-level clinical studies.

## 2 AVP

AVP is Apple's first head-mounted spatial computing device (Figure 1), officially released on June 6, 2023, and launched in the United States on February 2, 2024. The AVP device weighs around 600–650 grams and is powered by Apple M2 and R1 chips. The exterior major structure consists of a glass panel, with a 4 K ultra-high-resolution Micro-OLED display with a brightness of up to 5,000 nits and a screen resolution of 23 million pixels. It features an integrated 3D camera that can capture depth photographs and 3D videos. It is additionally equipped with 12 external cameras, a high-precision eye-tracking system, and a multi-sensor array, facilitating ultra-high-resolution and low-latency real-scene penetration and spatial positioning (8). The device's primary feature is the comprehensive integration of AR and VR technologies, facilitating real-time multi-screen data overlay and interaction with 3D models. It can seamlessly integrate preoperative imaging, intraoperative real-time data, and electronic medical records, providing an integrated solution for clinical decision-making and skills training.

**Figure 1:**
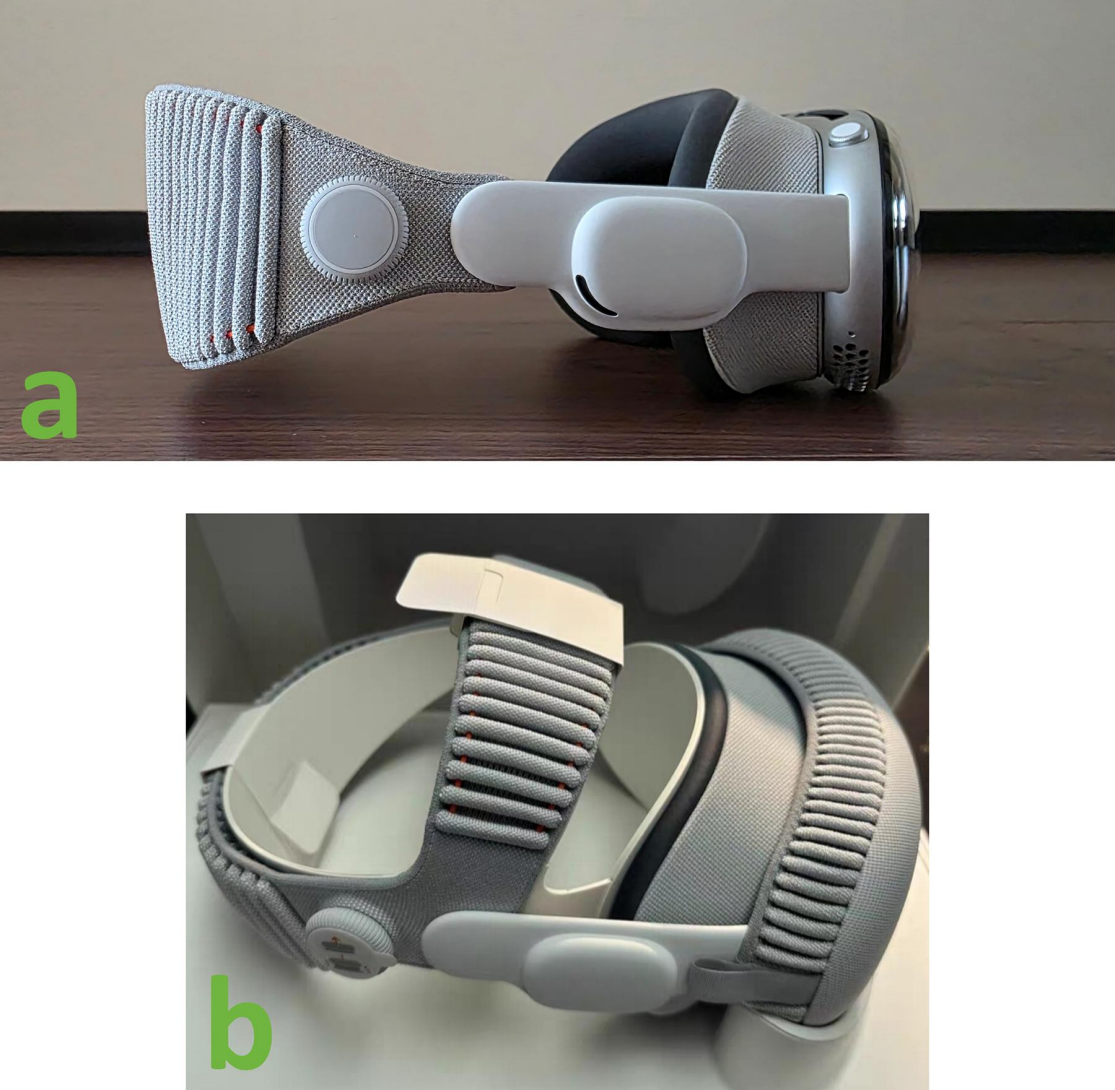
**(a)** Apple vision pro (2023 release). **(b)** Apple Vision Pro (2025 release).

The latest launched AVP in October 2025 is equipped with the advanced M5 chip, which significantly enhances chip performance and considerably enriches the spatial experience of the AVP (Figure 1). The display supports a maximum refresh rate of 120 Hz and renders 10% more pixels compared to the previous generation. The new version of AVP weighs about 150 grams more than its previous version, and its maximum battery life has been extended up to 3 h of general use. The headband adopts double-loop weaving design and is equipped with newly added counterweight.

## 3 Methods

To systematically evaluate the early applications of AVP in clinical and educational settings, we conducted a scoping review and reported it in accordance with the PRISMA-ScR guidelines. We searched for articles published from January 1, 2023, to September 9, 2026, in PubMed, Web of Science, Scopus, and the Chinese Medical Association (CMA) Journal Database. The search strategies were as follows: PubMed: “apple vision pro”[Title/Abstract]; Web of Science: (apple vision pro (Title) OR apple vision pro (Abstract)) AND 2023-01-01/2026-09-09 (Publication Date); Scopus: (TITLE (apple vision pro) OR ABS (apple vision pro)) AND PUBYEAR > 2022; Chinese Medical Association Journal Database: (Title, Keyword, and Abstract = apple vision pro) AND publication date = [2023-01-1 TO 2026-09-09]. The last search was performed on September 9, 2026. A total of 361 records retrieved from the four databases were exported to EndNote X9. Duplicates were first removed automatically using the “Find Duplicates” function, and the remaining records were manually screened by two independent reviewers (Yaqin Wang and Xiaohua Zuo) based on title, author, year, journal, and digital object identifier (DOI). Any disagreement regarding duplicate status was resolved by a third reviewer (Yiwei Hu). After deduplication, 251 records remained for content screening. Two independent reviewers (Yaqin Wang and Xiaohua Zuo) then performed relevance screening by reading the full texts. The inclusion criteria were articles involving the Apple Vision Pro and relevant to clinical medicine or medical education, while exclusion criteria were news reports, studies unrelated to the AVP product, and articles irrelevant to both fields. Any disagreement regarding inclusion was resolved by a third reviewer (Yiwei Hu). After screening, 2 articles were news reports, 180 were unrelated to the topic, and 69 articles were ultimately included in this review (Figure 2).

**Figure 2:**
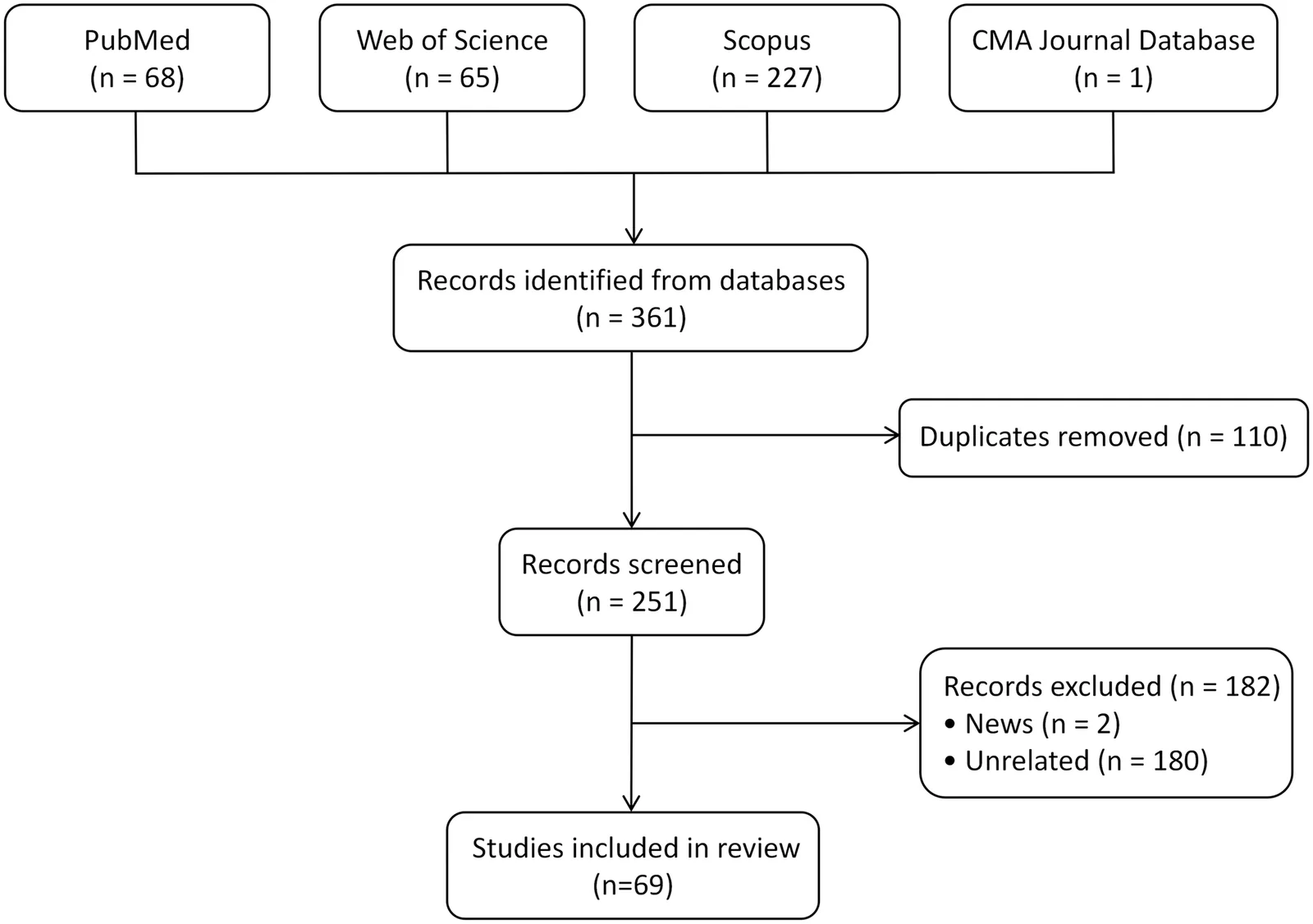
Flowchart of data collection.

To reflect the characteristics of study design and research setting, the included studies were categorized into three descriptive groups: 10 preclinical exploratory studies, 25 feasibility studies, and 34 medical education articles or opinion pieces. As a scoping review, we did not perform a formal risk-of-bias assessment or meta-analysis. Instead, we critically appraise the level of evidence using the Idea, Development, Exploration, Assessment, Long-term follow-up (IDEAL-D) framework (9). Stage 0: Preclinical exploratory studies, using phantoms, models, or *ex vivo* tissues as research subjects without involving real patients. Stage 1: First-in-human studies, involving a small number (typically ≤9) of real patients to preliminarily evaluate safety and technical feasibility (e.g., case reports, small case series). Stage 2a: Exploratory studies, involving a moderate sample size (typically ≥10) of real patients to assess initial effectiveness and optimize technical procedures (e.g., prospective case series, non-controlled trials). Stage 2b: Confirmatory studies, validating the effectiveness and safety of a device or technology through controlled designs (e.g., randomized controlled trials, multicenter controlled studies, large-sample diagnostic accuracy studies). Based on these criteria, we classified 10 studies as Stage 0, 23 as Stage 1/2a, 2 as Stage 2b. The two Stage 2b studies employed controlled designs with quantitative outcome measures, fulfilling the IDEAL-D definition of confirmatory studies. However, both were single-center studies with relatively small sample sizes and short follow-up, and neither evaluated long-term patient-relevant outcomes. Therefore, the overall body of evidence remains dominated by Stage 0–2a studies. The remaining 34 articles, comprising medical education studies without original patient data and opinion pieces, were not applicable to the IDEAL-D framework (Supplementary Table S1).

These two classification schemes serve complementary purposes. The three descriptive groups reflect study design and research setting, providing an overview of the evidence types available, while the IDEAL-D framework offers a standardized evidence-level stratification that enables direct comparison with the established medical device innovation pipeline.

## 4 Clinical applications

### 4.1 Types of MR applications

MR technology presents three main levels of application in clinical medicine: surgical navigation (MR-SN), image guidance (MR-IG), and image reference (MR-IR) (7). MR-SN uses real-time spatial tracking and dynamic registration to precisely track instruments and project their position onto preoperative images, enabling accurate virtual-to-anatomy matching and full procedural guidance—an advanced MR application. MR-IG provides intuitive visual assistance by converting medical images into 3D overlays on the surgical field, but instruments are neither tracked nor mapped. MR-IR statically displays imaging/anatomical information as a virtual floating reference, representing the simplest MR application in clinical medicine. Among the 35 studies classified within the IDEAL-D framework, none involved the application of an MR-SN system, 6 studies involved MR-IG systems, 26 studies described experience with MR-IR systems, and 3 studies did not involve any of the three techniques.

### 4.2 Preclinical exploratory studies

#### 4.2.1 Ultrasound-guided and percutaneous puncture studies

Several phantom studies have evaluated AVP for ultrasound-guided and percutaneous puncture tasks. Jacob et al. assessed AVP-assisted CT-guided percutaneous biopsy using an abdominal phantom and found that AVP improved puncture performance in less experienced operators, with a success rate of 36.4% versus 9.1% for conventional methods in complex targets, and reduced puncture time to 56.9% of conventional operation (10). In a similar line of work, Bexten et al. and Miller et al. reported favorable feasibility and usability for AVP-assisted ultrasound-guided central venous catheter placement and vascular access, with high puncture success rates and positive ratings for image quality and workflow, although user satisfaction varied (11, 12). Tregobov et al. further compared AVP-assisted needle placement with conventional sonography in a gelatin phantom, in which three experienced radiologists achieved 96% first-pass accuracy with AVP versus 100% with conventional sonography, alongside high ratings for hand-eye coordination and overall satisfaction (13). By contrast, Iribar-Zabala et al. compared HoloLens 2, Magic Leap 2, and AVP in MR-guided percutaneous puncture navigation, and while AVP achieved the smallest distance error during successful punctures (6.34 mm) and a SUS score of 70.17, its overall success rate was only 52.46% (14).

#### 4.2.2 Endoscopic and ergonomic studies

Beyond puncture tasks, AVP has also been explored in endoscopic simulation and ergonomic assessment. Okachi et al. developed an AVP-based navigational bronchoscopy simulation training system, in which two experienced pulmonologists achieved 80% success in target segmental bronchus access with a mean operating time of 85.9 s (15). In a related ergonomic study, Nagano et al. assessed flexible endoscopic procedures using an *ex vivo* porcine stomach model and found that the immersive AVP setting significantly reduced procedure time and NASA-TLX mental workload compared with non-ergonomic settings, and also reduced muscle activation in several neck and shoulder muscles (16).

#### 4.2.3 Navigational and positioning studies

AVP has also been applied to navigational and positioning tasks. Kawaguchi and Yamasaki developed an AR-guided fNIRS probe positioning system using AVP, with positional errors of 9.6 ± 2.9 mm and 7.6 ± 0.8 mm for the left and right primary motor cortex, respectively (17). Similarly, Ma et al. evaluated AVP-assisted guide plate positioning in lumbar surgery using preoperative CT, 3D-printed phantoms, *ex vivo* porcine specimens, and live porcine surgery (18). In phantoms, mean alignment error was below 3 mm, and in two live pigs, 12 pedicle screws were placed with mean angular and entry point errors of 1.29° ± 1.09° and 2.34 ± 1.37 mm, respectively (18).

#### 4.2.4 Suturing and fine motor tasks

Javaheri et al. examined whether AVP could support fine manual tasks (19). Using a within-subject controlled design, 20 medical staff completed suturing tasks while wearing AVP, and expert-assessed suturing quality showed no statistically significant difference compared with baseline without the headset (74.5 vs. 73.8), suggesting that AVP has the potential to support fine manual tasks without compromising precision (19).

Collectively, these preclinical studies suggest that AVP provides adequate spatial accuracy, preserves fine motor performance, and may offer ergonomic benefits. Building on this preclinical foundation, multiple investigators have progressed to real-patient studies to explore the practical feasibility of AVP in multidisciplinary surgical settings.

### 4.3 Feasibility studies

#### 4.3.1 Preoperative device connectivity

The techniques for connecting the AVP to laparoscopic and endoscopic devices are essentially identical, although slight differences might exist among various device models. Shen et al. described a connection method that was successfully applied in laparoscopic surgery (Figure 3): a HAGiBiS capture card was connected to the video output of the laparoscope, and the output of the capture card was linked to a MacBook via an USB-C interface, enabling real-time transmission of laparoscopic video. The OBS capture software was then run on the MacBook, and the resolution was adjusted to 960 × 740 to alleviate the video processing capacity and minimize latency. Under default settings, the latency was approximately 120 ms, which could be improved to under 100 ms with resolution adjustment (20). Following completion of the device connection, the surgeon equipped the AVP headset, performed surgical hand antisepsis, and proceeded with the operative procedure.

**Figure 3:**
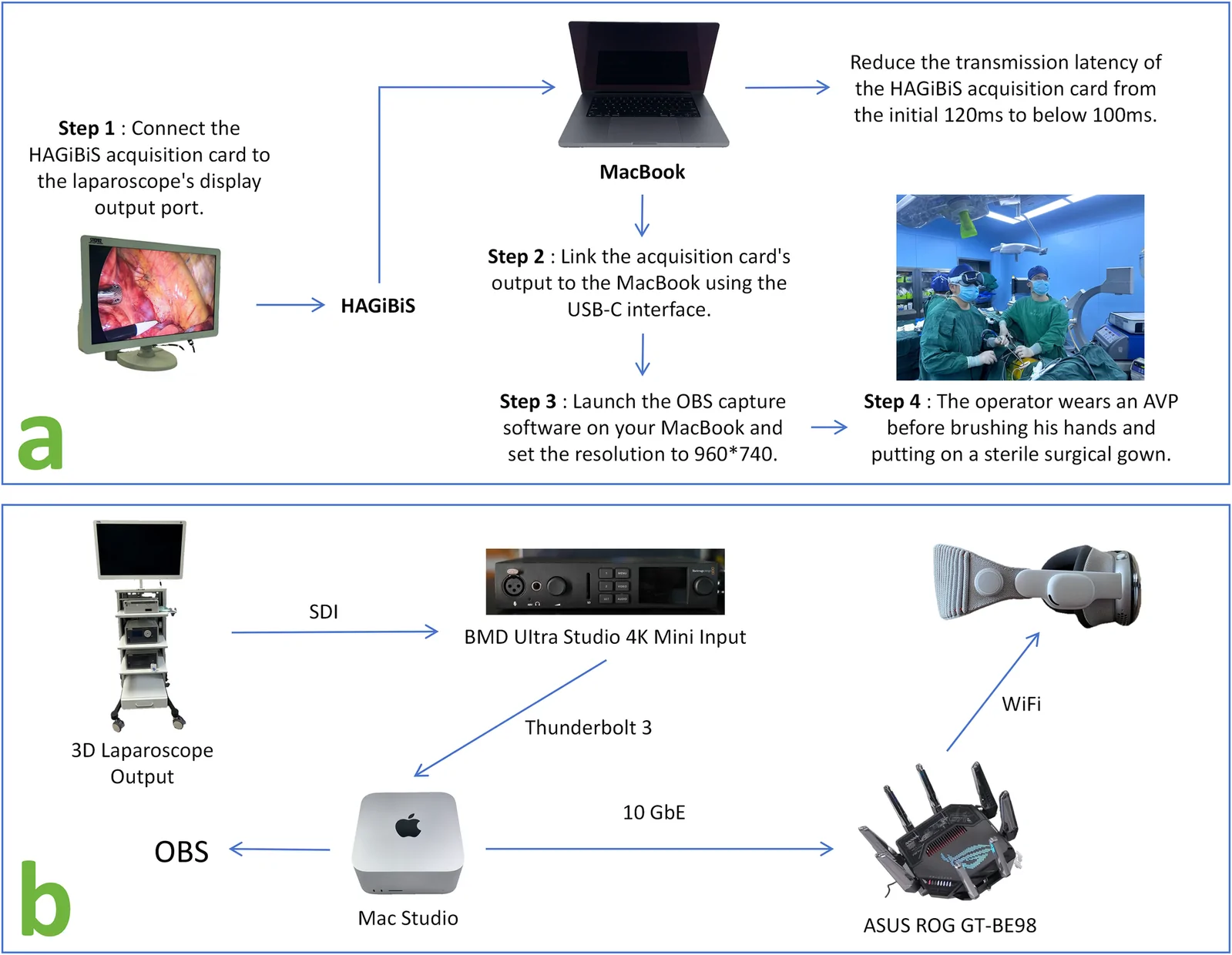
**(a)** Initial device interconnection setup. **(b)** Optimized interconnection setup.

Shen et al. conducted performance optimization of the system to further reduce latency (Figure 3): (1) Architecture of device communication: The SDI video signal from the 3D laparoscope was output to the SDI input of a Blackmagic Design (BMD) Ultra Studio 4 K Mini capture device, which was connected at high speed to a Mac Studio via a Thunderbolt 3 interface. The Mac Studio was linked to an ASUS ROG GT-BE98 router via a 10 GbE Ethernet connection, while the AVP connected to the same network via WiFi, thereby achieving interconnection and interoperability with the host device. (2) Software optimization strategy: The OBS capture software was deployed on the Mac Studio to provide precise resolution matching with the output signal of the 3D laparoscope. The system supported two high-resolution output formats: 1080p at 60 fps or 4K at 30 fps, which could be dynamically adjusted via software algorithms. The transmission latency was controlled to within 60 ms, allowing surgeons to observe a highly synchronized real-time 3D surgical field through the AVP (21).

#### 4.3.2 Laparoscopic/endoscopic surgery

With its core advantages of low-latency spatial computing, multimodal information integration, and 3D visualization, AVP has been deeply integrated with laparoscopy, cholangioscopy, and endoscopy, providing important technical support for minimally invasive procedures in spinal surgery, general surgery, interventional radiology, and neurosurgery. By integrating real-time intraoperative views with preoperative images and clinical data, it optimizes the ergonomics of surgical procedures, improving precision, safety, and efficiency.

In spinal endoscopic surgery, Park et al. combined AR with spinal endoscopy for lumbar stenosis (22). Real-time endoscopic video was displayed at the center of the AVP field, alongside electronic medical records and preoperative MRI, allowing surgeons to access information without diverting gaze. This reduced cervical strain, improved ergonomics, and enhanced decision-making safety. The video maintained ultra-high resolution without perceptible latency. The patient's lower limb pain disappeared immediately after surgery. At the 3-month follow-up, the visual analogue scale (VAS) score decreased from 6 to 1 for back pain and from 9 to 0 for leg pain. The Oswestry Disability Index (ODI) dropped from 52% to 12%, with no complications observed (22). On this basis, the same team later conducted a larger prospective study in 40 patients undergoing biportal endoscopic lumbar surgery (23). The results showed that the mean Surgery Task Load Index (SURG-TLX) cognitive load score of the operators after using the AVP was 22.24 ± 7.46 (out of a maximum of 100), indicating low mental burden. The incidence of intraoperative complications was low (two cases of dural tear without sequelae). At 3 months postoperatively, VAS and ODI improved significantly, comparable to conventional surgery, suggesting no adverse effect on early outcomes (23). This larger study indicates the feasibility and preliminary safety of AVP, with no increase in cognitive load, supporting wider use in minimally invasive spinal surgery. Furthermore, Bathini et al. used AVP in uniportal full-endoscopic resection of a lumbar facet cyst (24). Precise cyst resection and nerve root decompression were achieved, with immediate symptom relief sustained at 4 weeks and 3 months (24). This case further expands the scope of AVP application in endoscopic spinal decompression surgery and preliminarily validates its favorable visualization capability in managing intraspinal space-occupying lesions. Hanks et al. also reported a case of L4-5 biportal endoscopic transforaminal lumbar interbody fusion (TLIF) performed with the surgeon wearing the AVP throughout the procedure (25). The patient, an 81-year-old male with Grade 1 spondylolisthesis and lumbar stenosis, achieved complete resolution of radicular leg pain (VAS 7/10 to 0/10) and improvement of ODI from 14% to 2% at 2 months postoperatively. These improvements were maintained through the 7-month follow-up, with no intraoperative or postoperative complications reported (25). This case suggests that MR integration may be compatible with more complex reconstructive spinal procedures.

In minimally invasive general surgery, Broderick's team at the University of California, San Diego, USA, employed the AVP as the primary monitor to conduct 41 minimally invasive general surgeries (26). During surgery, clinicians could view up to three virtual displays simultaneously and adjust their position and size according to individual preferences. The mean NASA Task Load Index (NASA-TLX) score for the entire cohort was 22.3 ± 4.7, indicating low subjective cognitive load during surgery, with no significant difference between attending surgeons and trainee physicians (19.8 ± 5.3 vs. 24.8 ± 3.0, *p* = 0.23). No intraoperative complications occurred, and the 30-day morbidity and mortality rates were both 0, suggesting preliminary safety and usability of the AVP in this small series (26). Multi-screen functionality allowed side-by-side laparoscopic and endoscopic views without moving equipment, benefiting steps requiring intraoperative upper gastrointestinal endoscopy cooperation, such as esophagojejunal anastomosis. These findings provide preliminary evidence for expanding the application of AVP in laparoscopic and endoscopic surgery. Shen et al. further applied the AVP in 22 laparoscopic colorectal cancer surgeries, including malignancies of the ascending colon, sigmoid colon, and rectum (20). Surgeons wore the AVP throughout the procedures. The mean operative time was 189 min, and no intraoperative complications occurred. The SUS scores, stratified by surgical difficulty, ranged from 90 to 100, indicating that the AVP has good usability and safety in laparoscopic gastrointestinal surgeries of varying complexity (20). At Renji Hospital, AVP was combined with da Vinci robot and endoscopy for total gastrectomy, enabling concurrent visualization of tumor margins, instruments, and robotic arms, improving margin safety, console ergonomics, and operating space (27).

In interventional radiology, the AVP has been applied to cholangioscopy-guided biliary interventions. Zahra et al. reported a pilot case series of four biliary procedures performed by a single interventional radiologist wearing the AVP as the primary visualization device for real-time cholangioscopic video (28). All four procedures were technically successful without complications. The median procedure time was 105.0 min, while the median fluoroscopy time was 9.4 min. Qualitative operator feedback suggested improved procedural continuity and reduced need for repetitive head movement between monitors (28). This preliminary study suggests that AVP-assisted cholangioscopy can be safely applied in biliary interventions and may improve operator comfort.

In neuroendoscopic surgery, which requires great precision, the advanced eye-tracking and spatial computing capabilities of the AVP offer essential assistance for procedures. Olexa et al. applied AVP to neuroendoscopic procedures including spinal dural arteriovenous fistulae and ventriculoperitoneal shunt surgery (2, 29). The combination of preoperative DYNA CT/imaging data with the intraoperative endoscopic image facilitated 3D visualization of lesions, osseous structures, and intracranial anatomy. It allowed the surgeon to accurately manage spatial anatomical linkages and plan surgical trajectories, effectively minimizing the risk of intraoperative neurovascular injury (2, 29).

A late-breaking abstract further reported the feasibility of AVP in the bronchoscopy suite (30). In 30 interventional pulmonary procedures encompassing rigid, flexible, and robotic bronchoscopy, real-time procedural video was displayed within the operator's visual field. Mean NASA-TLX scores decreased from 42.5 ± 16.6 with conventional visualization to 30.1 ± 21.3 with AVP (*p* = 0.041), with consistent reductions across all procedural modalities. Setup time averaged five minutes, and operators reported improved visualization alignment and reduced neck strain (30).

#### 4.3.3 Ophthalmology

In the field of ophthalmic surgery, Orione et al. were the first to apply the AVP in corrective surgery for eyelid malposition (31). Ten ophthalmologists completed the procedures while wearing the AVP throughout the surgery. Using the AVP, the surgeons overlaid preoperative patient images in real time on the right side of the surgical field, while the left side simultaneously displayed information such as a surgical step checklist, the local anesthetic effect curve, and a timer, achieving integrated presentation of multi-source data. SUS scores all exceeded 85, and ratings for practicality, operational freedom, workflow integration, and educational value were all above 8.5 (out of a maximum of 10). No complications occurred. Although the scores for visual comfort and information clarity were relatively low (6.0 and 4.9, respectively, out of 10), Orione et al. suggested that this was mainly related to the operators' initial unfamiliarity with the device (31). This study suggested the feasibility of AVP in improving visualization, enhancing operational precision, and facilitating team communication in ophthalmic surgery.

Mastropasqua et al. further extended the application of AVP to more delicate intraocular procedures, prospectively enrolling three patients undergoing phacoemulsification with intraocular lens implantation, three undergoing silicone oil removal, and three undergoing vitrectomy combined with epiretinal membrane peeling (1). Using the multimodal image fusion function of the AVP, the surgeons could retrieve preoperative fundus autofluorescence, optical coherence tomography (OCT), and fundus photography images at any time during surgery. All procedures were completed successfully, with a 0% intraoperative and short-term postoperative complication rate. Surgeons rated their satisfaction with the integrated system in terms of controllability, workflow integration, and real-time information updating, all above 8/10. This study also explored an AVP-based remote surgical guidance model, connecting five trainee physicians at varying distances (from the same hospital to international hospitals) via the FaceTime platform to share the surgical field of view in real time. No significant signal latency was observed throughout the process, and all trainees expressed satisfaction with the remote teaching model (ratings > 8/10), stating that it effectively filled knowledge gaps and enhanced surgical immersion (1).

Building on these preliminary experiences, Korn et al. conducted the first prospective study evaluating AVP as the primary visualization platform throughout complete cataract surgery in 45 patients (32). The AVP received a live stereoscopic feed from a ZEISS ARTEVO 850 digital microscope via a dedicated wireless network, with conventional microscope oculars and an external 3D monitor available as backup. Forty-four of 45 procedures were completed without device-related conversion. One case required backup conversion after a transient image interruption, with no patient harm. No device-related complications occurred. Best-corrected visual acuity improved from 0.50 to 0.02 logMAR at 1 month (32). Although the study did not include objective latency or ergonomic measurements and thus cannot establish comparative safety or routine clinical suitability, the results nevertheless indicate that AVP-based visualization may be feasible when used in a backup-supported setting.

El Jalbout et al. further evaluated AVP as the sole intraoperative display for endoscopic dacryocystorhinostomy (endoDCR) in a consecutive, non-randomized study of 32 patients (16 AVP vs. 16 traditional monitor) (33). AVP was associated with shorter operative time (34.4 ± 8.6 vs. 42.7 ± 8.5 min; *p* = 0.006) and lower overall NASA-TLX workload (median 19.2 vs. 40.0; *p* = 0.031), with 100% functional success and no intraoperative complications in both groups. The authors estimated a modeled saving of approximately $420 per case (33).

Together, these studies suggest that AVP has acceptable clinical feasibility and safety in both eyelid and intraocular surgeries, and its multimodal image overlay and remote guidance functions provide innovative tools for precision ophthalmic surgery and surgical training.

#### 4.3.4 Ultrasonography

In the field of ultrasound-guided interventional procedures, puncture accuracy directly influences treatment efficacy. By employing multimodal image fusion and real-time 3D visualization technology, AVP has improved the precision and efficiency of liver tumor ablation. Tsai et al. were the first to use AVP in a case of laparoscopic ultrasound-guided radiofrequency ablation of a liver tumor (34). In this case, the probe localization time from skin to tumor center was only 41 s, representing an approximately 30% improvement in tumor localization efficiency compared with conventional methods (typically over 1 min). Furthermore, the procedure eliminated the need for a circulating nurse to assist with image management, enabling smooth and precise ablation (34).

On this basis, Lan et al. developed an AVP-based real-time image fusion navigation system that integrates the laparoscopic view, intraoperative ultrasound, and a 3D liver model into the operator's natural field of view (35). In a controlled phantom study, this navigation system increased the accuracy of hepatic hemangioma ablation puncture from 42.5% to 90%, representing an improvement of 47.5%. Specifically, the success rate improved from 15% to 80% for less experienced physicians and from 70% to 100% for senior physicians. The NASA Task Load Index showed that the system significantly reduced the operators' mental, physical, and temporal workload (all *P* < 0.05). Lan et al. then used this system to perform ablation in eight patients with hepatic cavernous hemangiomas. All ablations were successful and no intraoperative complications occurred (35).

These two studies, ranging from phantom experiments to real patient surgeries, provide preliminary evidence that AVP can integrate ultrasound, laparoscopic views, and 3D imaging, thereby optimizing puncture accuracy and operational efficiency while reducing cognitive load across operators with different experience levels. These findings provide a new technical solution for ultrasound-guided precision interventional therapy.

#### 4.3.5 Urology

Montoro Neto et al. were the first to apply AVP in ultrasound-guided mini-endoscopic combined intrarenal surgery (Mini-ECIRS) (36). Using the multi-screen display function of the AVP, the operator simultaneously observed the views from the nephroscope and flexible ureteroscope, while projecting the ultrasound image as a virtual screen into the field of view, thereby avoiding the line-of-sight obstruction caused by equipment in conventional operations and improving puncture ergonomics and operational efficiency (36).

Subsequently, Wen et al. applied AVP in retrograde intrarenal surgery (RIRS) for a patient with bilateral renal stones and a right ureteral stone (37). During the procedure, the operator used the AVP to simultaneously display the surgical field, real-time C-arm images, preoperative plain kidney-ureter-bladder (KUB) radiography, computed tomography (CT), and 3D reconstruction models, achieving precise stone localization and efficient lithotripsy. The patient had no postoperative complications and was discharged the next day (37).

#### 4.3.6 Radiology

In an initial investigation of the diagnostic performance of the AVP, Murphy et al. suggested that its primary application scenarios include the interpretation of abdominal/pelvic CT images and the 3D reconstruction and visualization of complex anatomical structures (38). The study indicated that the diagnostic efficacy of the AVP was comparable to that of conventional desktop monitors. For CT-based diagnosis of diverticulitis, the area under the curve (AUC) of the AVP was 0.93 (95% CI: 0.88–0.97), which was not statistically significantly different from that of desktop monitors (AUC = 0.94, 95% CI: 0.91–0.98; *p* = 0.40). The AVP was suitable for radiologists with varying degrees of experience. The AVP demonstrated significant advantages in immersion and intuitiveness when used for 3D reconstruction, with a mean preference score of 3.3 on a 5-point scale, which was significantly higher than that of conventional devices. In terms of operational experience, the ease-of-use score for the core functions of the AVP ranged from 3.3 to 4.7, with a low incidence of motion sickness and favorable eye-hand coordination. However, the device exhibited notable limitations: the median diagnostic time per case (57 s) was considerably longer than that of desktop monitors (31 s, *p* < 0.001); it lacked calibration for the Digital Imaging and Communications in Medicine (DICOM) Grayscale Standard Display Function (GSDF); physical comfort varied substantially among users; and operations such as window width adjustment were poorly adapted. As a result, radiologists still preferred conventional desktop monitors for basic image viewing scenarios (38). Overall, the AVP has advantages in the 3D visualization of complex anatomical structures.

This advantage was demonstrated in an emergency vascular case. Dandu et al. used AVP to generate a 3D volume-rendered model from preoperative CTA of an aorto-esophageal fistula (39). Preoperative CTA data were imported into the Visage Ease application on the AVP to generate a high-fidelity 3D volume-rendered model. Compared with 2D workstations, the AVP model more clearly showed esophageal stent erosion of the aortic wall, with natural gesture control and a short learning curve, improving spatial understanding (39). This case suggests that the spatial visualization capability of the AVP may not only facilitate diagnostic imaging but also provide more efficient support for preoperative planning and intraoperative decision-making in emergency situations.

#### 4.3.7 Orthopedics

In the field of orthopedic limb salvage surgery, Armstrong et al. were the first to apply the AVP in two complex limb salvage procedures for diabetic foot and Charcot arthropathy (40). The first case involved a 64-year-old female with Charcot arthropathy and medial tissue defect who underwent Ilizarov external fixator reconstruction. The second case involved a 62-year-old male with diabetic peripheral artery disease and toe gangrene who received antibiotic-loaded bone cement implantation and revascularization. During surgery, the operator used the AVP to simultaneously display preoperative 3D reconstruction images, angiographic images, and perfusion images as floating projections within the surgical field, thereby achieving real-time 3D understanding of complex skeletal deformities and vascular anatomy, and optimizing intraoperative decision-making and multidisciplinary team communication. The AVP was also used for remote guidance of overseas trainee physicians and for postoperative teaching debriefings using recorded videos. Although the authors noted that the current real-world mixed reality pass-through resolution of the AVP is insufficient to support highly dexterous fine manipulations, its value in understanding complex anatomical relationships, integrating multimodal imaging, and surgical education has been preliminarily validated, providing an important reference for the clinical translation of XR technologies in orthopedics, particularly in foot and ankle reconstruction and limb salvage surgery (40).

#### 4.3.8 Psychiatry and pain management

The AVP has also been preliminarily explored as a non-pharmacological tool for pain and anxiety management in hospitalized patients. Azar et al. reported the first case study using the AVP-delivered Mindfulness application in a 22-year-old female inpatient with mixed connective tissue disease, who presented with progressive acute-on-chronic pain and significant health-related anxiety that reinforced her as-needed hydromorphone use (41). Over a 48-hour period, the patient completed six mindfulness sessions, using the AVP's built-in Mindfulness application to guide her breathing through an expanding and contracting floral visual interface. Pain VAS scores decreased from a pre-intervention mean of 4.67/10 to 1.83/10 post-intervention (*P* = 0.03), while anxiety VAS scores decreased from 6.5/10 to 2.83/10 (*P* = 0.03). The patient reported high overall satisfaction (5/5) and a strong willingness to reuse the device (5/5), citing immersive visuals, calming voice guidance, the device's breathing pattern detection, and its ability to minimize external distractions as key advantages. Notably, although the patient had previously expressed interest in mindfulness but declined conventional therapist-guided interventions, the immersive AVP experience successfully facilitated her engagement, suggesting that VR/AR delivery may enhance adherence to mindfulness-based therapies (41).

#### 4.3.9 Thoracic surgery

In thoracic surgery, AVP has been investigated as a tool for spatial reorientation of resected specimens during staged chest wall reconstruction. This setting presents unique challenges, including wound bed deformation and loss of native anatomical landmarks, which complicate accurate margin localization and surgical decision-making. Radevic et al. reported a case using AVP for this purpose (42). A specimen-derived 3D model was generated and virtually annotated to mirror the pathological grossing process. The annotated model was then uploaded to AVP for holographic reorientation into the resection bed during staged chest wall reconstruction. The patient was a 71-year-old male with recurrent undifferentiated pleomorphic sarcoma of the chest wall. He underwent radical resection including the left chest wall and ninth rib, with planned reconstruction four days later. After resection and before formalin fixation, the specimen was scanned using a handheld structured-light 3D scanner. The resulting digital twin was annotated within a custom computer-aided design software to replicate inked surfaces, cut sections, and cassette labels corresponding to the final pathology report. On the day of reconstruction, the annotated 3D model was uploaded to the AVP. The surgeon successfully reoriented the holographic specimen model into the wound bed using the resected rib margin and inked surface orientations as fiducial landmarks (42). This report preliminarily confirms that AVP-based holographic specimen reorientation is compatible with routine surgical and pathological workflows. It also provides a framework for targeted margin relocalization.

#### 4.3.10 Preoperative informed consent

An emerging area of investigation concerns the impact of AVP on the patient-physician relationship. This dimension is critically shaped by eye contact, which strongly influences patient perceptions of physician empathy and trust. Chriqui et al. conducted the first RCT to evaluate whether anesthesiologists wearing the AVP during preoperative informed consent affected patients' perceived compassion (43). In this study, 60 patients each completed two encounters with the same anesthesiologist in randomized order, one with and one without the AVP (with EyeSight activated). The validated Sinclair Compassion Questionnaire (SCQ) yielded significantly lower scores in the headset condition (4.42 vs. 4.69, SD 0.87 vs. 0.48, *P* = 0.003), with the largest decline observed in eye contact satisfaction (*P* < 0.001, *r* = 0.736) and patient focus (*P* < 0.001, *r* = 0.614). While 53.3% of patients found EyeSight realistic, 25% opposed fully occlusive headsets without simulated eye contact. Despite general openness to healthcare technology, with 85% agreeing that technology eases physicians' work, over half of the participants (51.7%) believed explicit consent should be obtained before headset use (43).

These findings indicate that AVP may inadvertently undermine patient engagement and trust in face-to-face encounters, an effect not fully addressed by EyeSight. This highlights the need for careful implementation and, when indicated, informed consent.

### 4.4 Clinical application prospects

#### 4.4.1 Interventional radiology

AVP has been explored for preprocedural planning in interventional oncology. Zahra et al. conducted a pilot study in which 50 transarterial hepatic procedures were reviewed using the AVP with Visage Ease VP software prior to intervention (44). The median planning time was 5.0 min, representing approximately 6% of total case duration. AR/VR review changed the planned procedural approach in 46% of cases. The most common changes were improved identification of tumor-feeding arteries or clarification of complex hepatic arterial branching (61%), followed by changes in microcatheter position (30%) and selection (9%). Likert scores were high for anatomic visualization (4.7/5), anatomic understanding (4.7/5), procedural confidence (4.6/5), and workflow integration (4.8/5) (44). These findings suggest that AVP-based preprocedural planning is feasible and efficient, and may enhance anatomic understanding and procedural confidence with minimal workflow disruption.

Beyond oncologic applications, similar AVP-based AR assistants are being explored for general interventional radiology workflows. Xu and Wu co-designed PRISM, an AVP-based multimodal AR assistant, with two interventional radiologists (45). PRISM consolidated multi-screen imaging into an in-field XR workspace and used voice for discrete commands and hand gestures for continuous navigation under sterile conditions, with a phase-specific guided view for needle insertion (45).

#### 4.4.2 Plastic surgery

Dhawan et al. reported that AVP enhanced surgical decision-making and improved patients’ medical experiences to support the usefulness of AVP in plastic surgery (46). Preoperative planning with patient-specific 3D models improves efficiency and safety. Intraoperative AR overlay overcomes limitations of non-immersive devices, benefiting delicate procedures like rhinoplasty. VR also reduces preoperative anxiety and helps patients set realistic expectations, improving doctor-patient communication (46).

#### 4.4.3 Ophthalmology

Leveraging its high-resolution display, wide field of view, and multi-sensor fusion, AVP offers new possibilities for ophthalmic diagnosis, therapy, and rehabilitation (3, 47). It enables precise vision screening and low-vision assistance (e.g., night blindness), making it a portable alternative for large-scale blindness prevention in resource-limited areas (47). Furthermore, the see-through appearance in AR mode can reduce the social stigma associated with traditional head-mounted assistive devices, thereby increasing patients' willingness to wear them. In the field of strabismus rehabilitation, deep integration of the strabismus screening algorithm EyeTurn and the eye exercise application MyEyeGym with the AVP platform could, through immersive interaction, enhance extraocular muscle training and potentially provide a non-surgical intervention pathway for some patients (48). Other potential uses include metamorphopsia suppression, neurovestibular rehabilitation, and astronaut eye monitoring. Masalkhi et al. further proposed the AVP as a potential head-mounted perimetry device for glaucoma and spaceflight-associated neuro-ocular syndrome (49). They argued that its portability, high-resolution display, and game-like interface could enable home-based visual field testing, reducing reliance on bulky conventional perimeters such as the Humphrey Visual Field Analyzer (49).

#### 4.4.4 Dermatology

Guirguis et al. proposed three core applications of the AVP in dermatology (50). First, it optimizes Mohs micrographic surgery by digitally overlaying histologic images to break down information barriers between pathology and surgery. Combined voice and gesture commands guarantee a sterile environment throughout procedures. Second, it utilizes an artificial intelligence-based clinical decision support system (CDSS) to accurately determine aesthetic injection sites. When combined with preoperative treatment outcome visualization, it improves therapeutic accuracy and doctor-patient communication quality. Third, it employs photogrammetry and image segmentation techniques to measure lesion changes in psoriasis. Its cost is only 1/70 to 1/7 of that of conventional whole-body mole mapping systems, facilitating tele-dermatology consultations in primary and remote areas (50).

Kaye et al. further elaborated AVP's role in Mohs surgery: preoperative 3D modeling, intraoperative margin guidance, and postoperative debriefing/remote teaching (51). These findings corroborate the aforementioned perspectives and collectively reveal the potential of the AVP across the entire chain of dermatologic surgery.

#### 4.4.5 Psychology

AVP supports emotion recognition, psychotherapy, and telepsychotherapy. Its multi-camera and sensor array enable detailed facial expression and eye-tracking analysis, providing high-fidelity data for affective research beyond conventional EEG (52, 53). In psychotherapy, realistic scene simulation enhances exposure therapy, while 3D environments and facial rendering improve remote interventions and patient adherence, compensate for the limited immersion of conventional video calls (53).

#### 4.4.6 Rehabilitation medicine

VR systems, with their immersive interactive environments and precisely designed therapeutic scenarios, offer benefits in the recovery of musculoskeletal function and post-traumatic rehabilitation (54). The AVP with high-precision optical tracking and motion capture systems facilitates real-time acquisition and quantification of joint kinematic trajectories and fine motor control data in patients, making it especially appropriate for upper extremity rehabilitation assessment and training (3, 55, 56). As reported by Paladugu et al., comparing AVP-derived motion data with normative parameters enables personalized rehab and faster recovery (56). Moreover, the AVP exhibits efficacy in mitigating postoperative complications. For instance, following rotator cuff repair, VR-guided targeted rehabilitation training after rotator cuff repair improves shoulder function (57, 58). In rural areas with limited healthcare resources, the convenient and flexible rehabilitation modality enabled by the AVP can also improve the accessibility of rehabilitation treatment (59).

#### 4.4.7 Digital pathology

In digital pathology, PathVis has been developed as a mixed-reality platform on the AVP for gigapixel whole-slide image (WSI) visualization and multimodal AI assistance. It replaces mouse navigation with eye gaze, hand gestures, and voice commands, and integrates a content-based image retrieval engine for similar-patient search and a conversational AI assistant for real-time interpretation. Preliminary qualitative feedback from pathologists and oncologists suggested that spatial side-by-side comparison of similar cases and hands-free AI querying may reduce context switching and cognitive load (60).

Although these prospects remain theoretical, AVP's technological foundation has preliminarily validated feasibility. Future studies should assess test-retest reliability, long-term safety, and equivalence to conventional methods to translate AVP into clinical practice.

### 4.5 Critical synthesis of cross-specialty findings

#### 4.5.1 Latency and hand-eye coordination

Video see-through latency is a fundamental determinant of surgical performance, yet reported values vary widely across studies due to heterogeneous measurement methodologies. In laparoscopic gastrointestinal surgery, Shen et al. reported an initial latency of approximately 120 ms, which was reduced to <100 ms through resolution adjustment (960 × 740 pixels) and further optimized to <60 ms following hardware upgrades (20, 21). Apple's own specification for the R1 chip cites a photon-to-photon latency under 12 ms, though this represents native passthrough performance rather than end-to-end video transmission from external surgical devices. The clinical relevance of these differences remains unclear: while the literature suggests that experienced operators can tolerate delays of approximately 100 ms for telesurgical tasks, no study has systematically quantified the impact of latency on fine motor tasks such as ophthalmic microsuturing or microvascular anastomosis when using AVP. Furthermore, owing to the absence of standardized latency measurement protocols for assessments at the capture card, processing workstation, wireless network, and headset display, direct cross-study comparisons cannot be reliably conducted.

#### 4.5.2 Cognitive workload

Despite the heterogeneity in surgical specialties and task complexity, cognitive workload assessments have yielded remarkably consistent results. Broderick et al., in the first series of 41 minimally invasive general surgery cases using AVP, reported a mean NASA-TLX score of 22.3 ± 4.7, indicating a low perceived intraoperative workload. No significant difference was observed between attending surgeons and trainees (19.8 ± 5.3 vs. 24.8 ± 3.0, *p* = 0.23) (26). Similarly, in a prospective study of 40 patients undergoing biportal endoscopic lumbar surgery, Hanks et al. reported a mean SURG-TLX score of 22.24 ± 7.46. Consistent findings across two separate cognitive workload metrics (NASA-TLX and its surgical adaptation SURG-TLX), different surgical disciplines (general surgery and spine surgery), and operators of differing experience levels suggest that AVP imposes no excessive cognitive burden in routine procedures (23). However, several important caveats warrant attention. First, both studies predominantly involved highly experienced and skilled senior surgeons. Whether novice or surgeons in low-resource settings would report similar scores remains unexplored. Second, these assessments captured short-term workload after individual procedures, not the cumulative fatigue from prolonged or repeated use. Reports of mild dizziness after 2 h of wear and user complaints of excessive weight indicate that ergonomic fatigue may confound outcomes during prolonged or sequential procedures; this effect cannot be captured solely via post-procedure TLX assessments. Third, the low cognitive workload may reflect the relatively undemanding nature of MR-IR (image reference) applications, which constituted the majority of included studies, rather than the intrinsic usability of the device for more complex MR-SN (surgical navigation) tasks.

#### 4.5.3 Contradictory evidence in puncture accuracy

Perhaps the most instructive contradiction arises from two phantom studies of percutaneous puncture navigation. In a direct comparative benchmark of HoloLens 2, Magic Leap 2, and AVP for sacral nerve stimulation needle insertion, Amaia Iribar-Zabala et al. found that AVP achieved the smallest distance error during successful punctures (6.34 mm) but an overall success rate of only 52.46%, with lateral errors in failed attempts reaching 19.39 mm. User feedback cited unstable marker tracking, excessive weight, and incompatibility with prescription glasses as major obstacles (14). By contrast, Tao Lan et al., using an AVP-based real-time image fusion navigation system for laparoscopic microwave ablation of hepatic hemangiomas, reported a puncture success rate of 90% under AVP guidance compared with 42.5% using conventional methods (35). This discrepancy is not a failure of the AVP hardware *per se*, but rather reflects fundamental differences in the navigation paradigm. Amaia Iribar-Zabala's study employed optical marker tracking for dynamic instrument guidance—an MR-SN (surgical navigation) approach that requires continuous real-time registration of the needle tip to the virtual target. This paradigm is highly sensitive to headset displacement, tracking drift, and line-of-sight occlusion, all of which are exacerbated by the AVP's weight and fit instability. Tao Lan's study, in contrast, employed static image fusion—an MR-IR (image reference) approach that overlays preoperative 3D models and intraoperative ultrasound onto the surgical field without tracking the ablation needle. This paradigm places lower demands on spatial registration stability and is more forgiving of headset movement. The contrasting outcomes thus suggest a critical hierarchy of AVP suitability: the device currently performs well for MR-IR applications (static image reference) and moderately for MR-IG (image guidance with 3D overlay), but remains immature for MR-SN (dynamic surgical navigation with instrument tracking). This interpretation aligns with the observation that, among the 35 studies classified within the IDEAL-D framework in the present review, none involved MR-SN, 4 involved MR-IG, and 23 described MR-IR experiences—a distribution that reflects the technology's current “safe zone” rather than its ultimate potential.

#### 4.5.4 Separating feasibility from clinical benefit

To avoid conflating technical feasibility with clinical benefit, we categorized the extracted outcomes into four domains: technical results, usability/ergonomics, operator performance, and patient-level clinical benefit (Table 1). This distinction is important because the ability of AVP to display images, integrate multiple data sources, or reduce head movement does not automatically translate into improved patient outcomes. While technical and operator-level metrics were frequently reported, patient-level clinical benefit remained sparsely evaluated. Overall, the current evidence mainly supports technical feasibility, usability, and operator-level performance. Evidence for patient-level clinical benefit remains limited and should be regarded as preliminary.

**Table 1:** Outcome domains for separating technical feasibility from clinical benefit.

Domain	Definition	Examples from included studies	Current evidence level	Interpretation
Technical results	Device connection, latency, image quality, tracking, system stability	Latency 60–120 ms; 4K display; wireless streaming; image fusion	Stage 0–2a	Feasible, but not standardized
Usability or ergonomics	Operator workload, comfort, usability, learning curve	NASA-TLX, SURG-TLX, SUS, neck strain, setup time	Stage 1–2a	Preliminary positive signals, short-term and small samples
Operator performance	Task accuracy, operative time, success rate, errors	Puncture accuracy, operative time, success rate	Stage 0–2a	Potential improvement, but heterogeneous
Clinical benefit or patient outcomes	Complications, VAS, ODI, functional success, patient-reported outcomes	VAS/ODI improvement, functional success, complications	Stage 1–2a	Limited; Requires controlled confirmation

## 5 Medical education applications

The AVP, as a next-generation spatial computing device, surpasses the limitations of conventional educational frameworks by combining VR and AR. It has been used in multiple areas including anatomical education, clinical skills training, specialized procedural simulation, and distant learning in medical education, offering technical support for the innovation of medical education. Moreover, it offers a novel strategy for tackling the issues of unequal resource allocation, low efficiency in skills and specialized training, and high costs in distance education.

### 5.1 Anatomical education

The AVP can overlay digital anatomical models onto real-world scenarios to supplant traditional cadaveric dissection teaching via generating highly realistic 3D anatomical environments using AR. This approach not only circumvents the issues of cadaver shortage, ethical controversies, and high costs but also enhances learners’ comprehension of intricate anatomical relationships through interactive manipulation—such as stepwise dissection of anatomical structures and dynamic demonstration of physiological functions (61). For instance, the INSIGHT HEART delivers a dynamic, stereoscopic, and dissectible cardiac model that vividly illustrates the entire cardiac pumping cycle, enabling learners to comprehend cardiac anatomy, rhythm, and blood flow patterns (Figure 4). Patil et al. developed a reproducible mixed-reality anatomy learning platform on the AVP that integrates the open-source Z-Anatomy atlas with a context-grounded retrieval-augmented generation (RAG) voice assistant bounded by Terminologia Anatomica (62). Using cardiovascular anatomy as the case domain, the system supports hand-gesture manipulation, eye-gaze selection, layer toggling, spatially anchored labels, and natural-language voice queries. These examples illustrate how AVP-based platforms can integrate open-source anatomical content with immersive interaction and AI-assisted tutoring to support anatomy education, although their educational impact remains to be validated.

**Figure 4:**
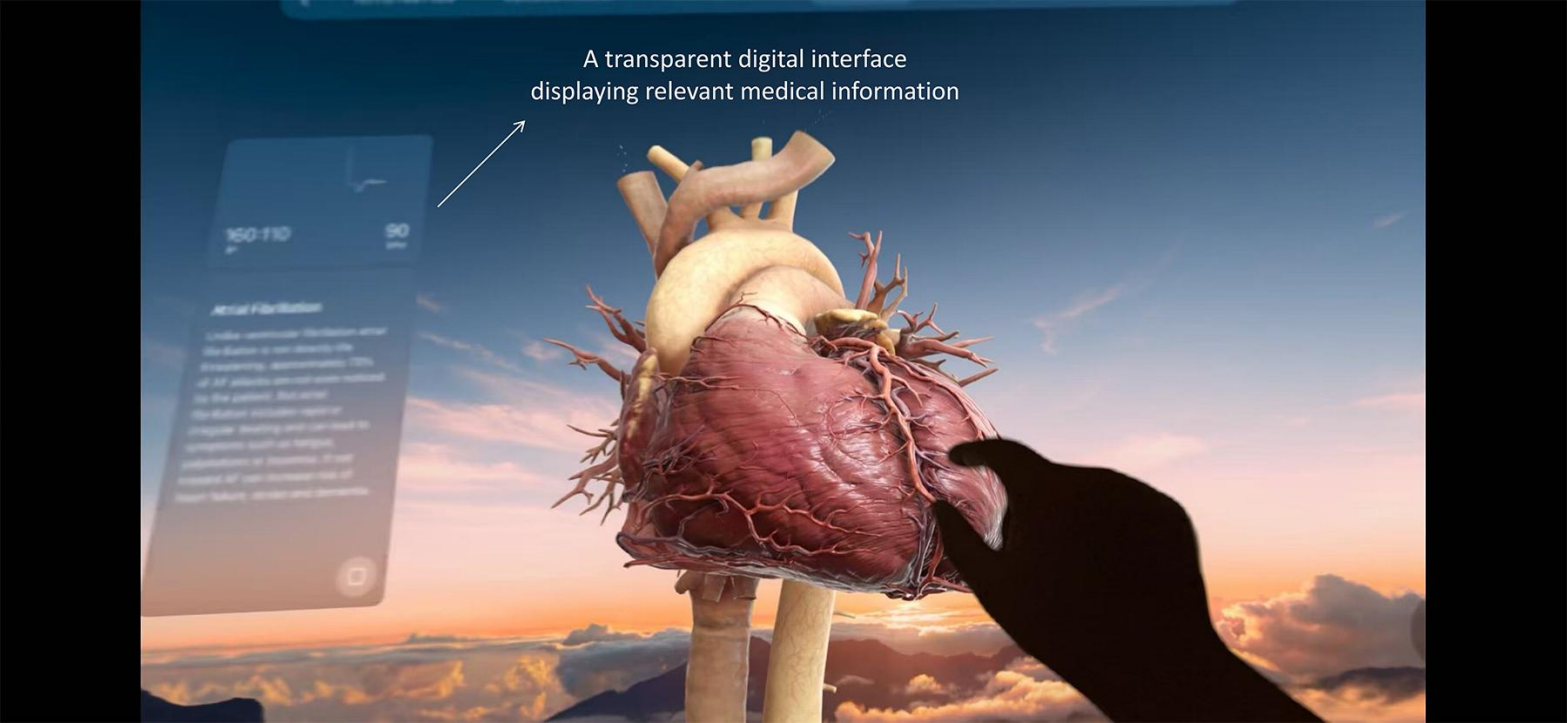
3D heart model from the INSIGHT HEART application on AVP.

### 5.2 Clinical skills training

The AVP improves the efficacy and safety of clinical skills training by establishing repeatable, low-cost simulation training scenarios through the use of VR (61, 63). Waisberg et al. proposed that the AVP could be used by surgeons to practice surgical skills in an immersive virtual environment, thereby improving patient outcomes (64). For ultrasound-guided regional anesthesia (UGRA), learners experience expert procedures with real-time 3D atlases, AI-enhanced layers, and remote ultrasound images, overcoming geographic barriers via bidirectional interaction (5). Multiple phantom studies support AVP's feasibility for CT-guided biopsy, suturing, CVC insertion, and navigational bronchoscopy, improving anatomical recognition and accuracy (10, 11, 14, 15, 19, 65).

In the field of emergency skills training, Jayawardene et al. developed a mixed reality naloxone training system based on the AVP (66). The team's initial VeNT system study (*N* = 25) validated its feasibility in overdose recognition, naloxone administration, and cardiopulmonary resuscitation training, with participants showing improvements in knowledge mastery and rescue confidence (66). After iterative upgrading to ReviveXR, two field tests (*N* = 25, with 20 participants completing both rounds) showed superior immersion and practicality over traditional methods (67).

Waisberg et al. further noted that the high-precision eye-tracking and gesture recognition technologies of the AVP can be used to construct an immersive virtual surgical environment, enabling learners to repeatedly practice complex procedures in a risk-free setting while allowing objective quantitative assessment of trainees’ technical competencies (68). This approach has the potential to transform the traditional apprenticeship model of surgical training. In laparoscopic education, Shen et al. reported that 50 trainees improved operative skills by 15%, exam scores by 9%, and 88% reported enhanced professional identity (21). A comprehensive review examined AI and MR technologies in orthopedic medical education, including the AVP as a leading MR platform. It reported high-resolution immersive visualization and workflow benefits in live surgery (69). In orthopedic surgical training, Stauffer et al. developed an AR-enhanced physical simulator for femoral nailing using the Apple Vision Pro as the display platform (70). The hybrid system combined a synthetic femur, real surgical instruments, and AR-based procedural guidance for nail insertion and rotational alignment. Twenty-three participants (6 experts and 17 non-experts) completed three unguided simulation runs followed by a retention session. Task completion time decreased by 47.1% for insertion and 57.4% for alignment across runs (both *P* < 0.001). The number of simulated fluoroscopic images declined by 45.6% for insertion and 49.2% for alignment. Implant placement accuracy remained stable, with insertion depth errors around 2-3 mm and rotational alignment errors around 1–2°. Experts consistently performed faster than non-experts, supporting the simulator's construct validity (70). By combining AR-based guidance with physical models and real instruments, this approach enables unsupervised, repeatable practice while maintaining authentic tactile feedback.

### 5.3 Medical education in specialized disciplines

The AVP facilitates customized courses designed for various medical specializations. In neurosurgery, AVP converts preoperative images into 3D anatomical models for interactive visualization from the surgeon's perspective and records entire procedures for debriefing and case library creation, enhancing understanding of complex surgical workflows (29). Moreover, integration with Apple Intelligence, through AI-driven writing, image generation, and voice interaction, enables rapid creation of teaching resources and personalized learning, offering efficient support for surgical simulation and anatomical education (71).

Conventional dermatologic surgery often faces issues such as interference with the procedure due to camera operation and limited viewing angles (72–74). The AVP can capture high-fidelity first-person surgical views, clearly presenting anatomical relationships and procedural details, thereby enhancing the intuitiveness and efficiency of medical education (72). In Mohs micrographic surgery training, the AVP permits 3D postoperative surgical review, enabling learners to dissect resection steps from the surgeon's perspective and replicate practice across different tumor scenarios (50).

In ophthalmic education, AR technology can simulate the visual manifestations of ophthalmic diseases to enhance understanding of disease characteristics. Recorded surgical videos may further serve as standardized educational materials, supporting ophthalmologists in mastering delicate microsurgical procedures (1, 31, 47). In this context, the AVP has recently emerged as a novel platform for ophthalmic surgical education. The ZEISS Surgery Optimizer application, developed specifically for the AVP, enables 3D recording, archiving, and immersive playback of surgical videos, allowing trainees to review cases performed by experienced surgeons in a stereoscopic, microscope-like view. Such 3D video-based review may accelerate skill acquisition and improve comprehension of surgical anatomy (75). However, compared with established simulators such as Eyesi and HelpMeSee, which have demonstrated construct validity, the AVP-based ZEISS Surgery Optimizer currently lacks formal validation studies to assess its impact on surgical performance or clinical outcomes. In addition, Amirpour et al. used the AVP and Meta Quest 3 to view stereoscopic cataract surgery videos in six experienced surgeons (76). Stereoscopic viewing was reported to improve depth perception and spatial awareness for training and live procedure observation, although resolution and left–right alignment needed improvement (76).

Tomiuk et al. evaluated the AVP's eye-tracking capabilities for training in hybrid ventricular septal defect procedures (77). In a two-part study with 44 participants, the AVP achieved a combined spatial accuracy of 0.59° and RMS precision of 0.41° in a 2D random saccade task, and 3.70° accuracy and 0.96° RMS precision when selecting anatomical landmarks on a 3D pediatric heart phantom. The authors concluded that AVP eye tracking is promising for navigation guidance and surgical training, although accuracy decreased for small, closely spaced targets.

Luijten et al. evaluated the usability of Siemens' Cinematic Reality on the AVP for hepatobiliary CT and MRCP visualization in 14 medical experts, including 11 surgeons, one doctor assistant, and two medical students (78). The mean SUS score was 77.68, indicating good-to-excellent usability, with high ratings for suitability, conformity, and controllability. Participants identified education and training, preoperative planning, and case discussion as promising applications.

### 5.4 Distance education

The AVP's immersive remote interaction facilitates real-time bilateral mentoring between professionals and students (79). The combination of 5G networks and the AVP offers a novel technical solution for remote surgical education. Goh et al. utilized an indoor 5G small-cell architecture to transmit the high-definition 3D view from the da Vinci surgical robot during transoral robotic surgery (TORS) in real time to the AVP headsets of remote trainees via low-latency wireless transmission (mean latency: 22 ms) (80). Twenty trainees from seven countries obtained a stereoscopic, immersive viewing experience approximating the first-person perspective of the operator, and SUS score was 72, indicating good acceptability (80). This study provides preliminary evidence that an AVP-based live streaming system over a private 5G network can overcome the planar limitations of conventional surgical broadcasting, providing a reproducible technical framework for cross-regional, high-fidelity surgical education and real-time mentoring.

## 6 Comparison with similar products

### 6.1 Hardware specifications comparison

In addition to the AVP, the mainstream head-mounted MR devices currently available on the market include the Meta Quest 3, HoloLens 2, Magic Leap 2, and PICO 4. Head-mounted MR devices can be classified as direct (D-MR) or indirect (ID-MR). D-MR uses optical see-through lenses, allowing users to directly view the real environment with virtual content optically superimposed. Both HoloLens 2 and Magic Leap 2 fall into this category. ID-MR adopts video pass-through technology. It captures real-world scenes via cameras, digitally merges them with virtual elements, and renders the combined view on the display. AVP, Meta Quest 3, and PICO 4 are representatives of this category. Based on the above definition, the AVP is a typical head-mounted, video pass-through ID-MR device.

In terms of core specifications, the AVP leads its peers in resolution, field of view, and natural eye-hand-voice interaction. However, it is inferior to similar products interms of weigh and battery life. Table 2 compares the basic parameters of the above five products (Table 2).

**Table 2:** Basic technical specifications for mainstream head-mounted mixed reality devices.

Specifications	Apple Vision Pro	Meta Quest 3	HoloLens 2	Magic Leap 2	PICO 4
Type of MR	ID-MR	ID-MR	D-MR	D-MR	ID-MR
Release date	2024	2023	2019	2022	2022
Price	Starting at $3,499	Starting at $499	App. $3,500	Starting at $3,299	Starting at $430
Resolution (Pixels per eye)	3,660 × 3,200	2,064 × 2,208	2,000 × 1,500	1,440 × 1,760	2,160 × 2,160
Field of view	100 deg. horizontal	110 deg. horizontal, 96 deg. vertical	52 deg. diagonal	70 deg. diagonal	105 deg. (horizontal and vertical)
Interaction	Hand tracking, voice commands	Hand tracking,controllers	Hand tracking, voice commands	Hand tracking, controller, voice commands	Hand tracking, controllers
Eye tracking	Yes	No	Yes	Yes	Only in Pro/Enterprise variants
Weight	600–650 g	515 g	566 g	260 g (headset) + 420 g (compute pack)	586 g (headset only)
Battery life	Up to 2 h with external battery	Up to 2.2 h of general use	Up to 3 h of active use	Up to3.5 h of active use	Up to ∼3 h of active use

### 6.2 Clinical performance comparison

The video pass-through design of the AVP has inherent limitations. First, image quality depends on camera performance; the operating room's high-contrast scenes can cause overexposure or detail loss. Second, video signal processing causes latency. Even when optimized to under 60 ms, it still impairs hand-eye coordination. Third, near-field resolution and depth perception are poor, making close-up focusing difficult. These limitations are common to video pass-through technology. In a phantom puncture navigation study, although the AVP achieved the smallest distance error during successful punctures (6.34 mm), its overall success rate was only 52.46%, and the lateral error in failed attempts reached 19.39 mm. User feedback cited unstable marker tracking, excessive weight, and incompatibility with prescription glasses, resulting in significantly lower reliability than optical see-through devices for high-precision interventional navigation (14).

In contrast, optical see-through devices offer direct, latency-free perception of the real environment. The HoloLens 2 uses transparent holographic lenses, allowing unobstructed direct vision with full situational awareness (7). Its clinical value is established: a mixed-reality navigation study (20 intracranial patients) showed registration time of 5.7 ± 2.0 min vs. 9.3 ± 2.9 min for conventional systems (*P* < 0.001), with comparable accuracy. Nowak et al. used HoloLens 2 and Magic Leap 2 to successfully manage complex lacrimal duct obstructions (81). Meta Quest 3 has achieved clinically acceptable accuracy in DIEP flap navigation, and PICO 4 has been preliminarily applied in emergency skills training (82, 83).

Overall, optical see-through devices, such as HoloLens 2 and Magic Leap 2, remain the mainstream platform for intraoperative use due to their established clinical validation and compatibility with existing navigation systems, offering advantages in fine anatomical identification, real-time instrument tracking, and complex spatial perception. In contrast, the AVP and other video pass-through devices excel in display quality, hardware performance, and software ecosystem, making them more suitable for surgical education, preoperative planning, remote consultation, and patient communication—scenarios with less demand for real-time response and close-range manipulation. Rather than one category being universally superior, these devices appear to serve complementary clinical niches, and direct comparisons should be interpreted with caution given the heterogeneity of study settings and outcome measures.

## 7 Discussion

### 7.1 Product limitations

The inherent technical limitations brought by the video see-through design of the AVP are as described above. Moreover, the AVP weighs between 600 and 650 grams and has inadequate near-field resolution and limited depth perception. Clinical studies suggests that prolonged wearing often causes cervical fatigue in surgeons. Minor alterations in the position of the headset significantly impair the precision of eye-tracking, leading to discrepancies between virtual content and actual anatomical structures. Its battery life of roughly 2 h is insufficient to meet the need for lengthy surgical procedures. Although battery replacement or external power supply may act as compensating solutions, they substantially weaken surgical continuity and the surgeon's procedural flexibility (84).

Notably, most included studies were conducted based on the original AVP. Apple released an updated AVP in 2025 with enhanced performance, higher refresh rate and doubled pixel count, alongside a one-hour longer battery life. However, its weight also increased by 150 grams. These hardware modifications may affect previous research findings in multiple aspects. Firstly, the added weight reduces operational comfort for medical staff and exacerbates neck fatigue, which may contradict previous favorable outcomes. Secondly, the elevated refresh rate cuts down motion latency, potentially improving hand-eye coordination in see-through visual navigation. Additionally, extended battery duration supports longer surgical procedures, while the heavier weight limits prolonged wearability. Accordingly, conclusions drawn from the original Apple Vision Pro cannot be directly applied to the new version, and relevant findings require re-verification.

The clinical application software suitable for the AVP remains in an exploratory development phase. Currently, the connection between the AVP and medical devices such as endoscopes and laparoscopes is complex, and most scenarios rely on adaptation through general-purpose software. There is a lack of specialized application software for individual specialties, and supporting service systems including software updates, troubleshooting, and technical training are not yet mature. Furthermore, Apple's strategy regarding hardware iteration and system updates may affect the stability of long-term clinical applications, and the risk of device discontinuation or operating system upgrades causing incompatibility with existing medical applications objectively exists.

### 7.2 Social limitations

#### 7.2.1 Workflow and system integration

The compatibility of the AVP with existing hospital information systems (HIS), electronic medical records (EMR), and picture archiving and communication systems (PACS) is suboptimal, and the lack of standardized data interfaces makes seamless integration difficult. Additional equipment such as routers, computers, and capture cards must be deployed intraoperatively, increasing operational complexity and potential points of failure. Conventional surgical workflows heavily rely on physical facilities including fixed monitors, surgical lights, and instrument tables. After donning the headset, processes such as visual communication with the surgical team, sterile field manipulation, and rapid device removal in emergency situations require redesign. Most existing studies are led by technical teams and lack surgeon-centered workflow optimization research. If the AVP cannot be integrated into current operating room layouts and team collaboration models, its widespread clinical acceptance will be difficult to achieve.

#### 7.2.2 Regulatory status

AVP and its accompanying medical application software have not yet obtained marketing approval from major regulatory bodies such as the FDA or NMPA as diagnostic or surgical navigation devices (84–86). Most existing studies are feasibility or exploratory trials, and the device itself has not been included within the scope of medical device registration management. If clinicians use the AVP for diagnostic or treatment decisions without approval, they may face legal risks including unclear medical liability determination and inadequate patient informed consent.

#### 7.2.3 Diagnostic display validation

Medical diagnostic displays are normally required to comply with the DICOM Grayscale Standard Display Function (GSDF) and to undergo regular calibration for luminance, contrast, and color accuracy. The AVP has not been validated against these standards, and studies have reported that it lacks DICOM GSDF calibration and shows variable color and brightness performance. Therefore, its use for primary diagnostic interpretation cannot currently be recommended.

#### 7.2.4 Intraoperative safety and liability

Intraoperative reliance on a head-mounted display introduces several additional risks. Transient image interruption, latency, or disconnection may impair hand–eye coordination during fine surgical tasks; even when latency is optimized to below 60 ms in experimental settings, backup visualization systems and fail-safe protocols should be mandatory in any intraoperative deployment.

Wireless streaming of surgical video and patient data creates potential attack surfaces. Security standards for the AVP data pipeline, including encryption, access control, and audit trails, remain incompletely defined. Institutions should ensure isolated, secured networks and comply with local cybersecurity regulations. In addition, the AVP can collect, transmit, and store sensitive patient information, including intraoperative video and imaging data. Clear standards are lacking for cross-institutional data sharing, de-identification in multicenter studies, and the scope of patient informed consent for virtual imaging data. Data breaches could seriously violate patient privacy and expose institutions to legal and reputational risks.

Infection control is another concern. The AVP is not designed as a sterile or cleanable medical device. Repeated use in operating rooms raises concerns regarding surface disinfection, headset sharing, and contamination of the sterile field. Dedicated cleaning protocols and, where possible, single-user assignment are needed.

Finally, if intraoperative device failure contributes to patient harm, responsibility among the clinician, hospital, device manufacturer, and software developer remains unclear. Existing medical liability frameworks do not adequately address algorithmic or hardware-related errors. Clear guidelines and liability insurance mechanisms are required before routine intraoperative use. Future efforts should promote the registration and approval of the AVP and related software as medical devices, and establish corresponding clinical use guidelines and liability insurance mechanisms.

#### 7.2.5 Economic considerations

Economic considerations also pose a significant barrier. The AVP is priced at approximately $3,500, and hidden costs, including specialized software development, system maintenance, personnel training, technology updates, IT support, sterilization, and workflow redesign, will further increase the economic burden on healthcare institutions. Under the current medical payment system, AVP-assisted surgery has not yet been covered by any mainstream reimbursement system, leaving patients to bear the associated costs out of pocket. Moreover, high-quality health economics studies, such as cost-effectiveness or budget impact analyses, are absent, preventing comparisons with conventional methods. These challenges vary across healthcare systems: in commercially driven markets, device procurement may depend on demonstrated return on investment, whereas in publicly funded systems, regulatory and budget approval processes are often lengthy. For resource-limited primary hospitals, the accessibility of this device is low, which may exacerbate disparities in the distribution of medical resources. Until these economic and systemic factors are systematically addressed through implementation research and formal health technology assessments, large-scale clinical integration of the AVP will remain limited.

### 7.3 Evidence gaps and future directions

Throughout this review, we have deliberately used cautious wording to reflect the exploratory nature of the available evidence. Statements such as “suggests feasibility” or “preliminary evidence” are not intended to diminish the potential of AVP, but to accurately represent the current stage of clinical translation. Consistent with this, the main limitation of this review is the generally low level of evidence of the included studies, with few confirmatory studies. Most of the available studies are single-center technical demonstrations, lacking standardized operating protocols and uniform outcome measures, resulting in limited reproducibility and external validity. The current application scenarios are still in the feasibility exploration stage, have not been validated in routine clinical workflows, and lack long-term follow-up of patient outcomes as well as rigorous cost-effectiveness evaluations. Thus, the summary of AVP medical applications presented in this review should be regarded as an aggregation of early exploratory progress rather than definitive clinical guidance evidence. Given the predominance of Stage 0–2a evidence, clinicians and policymakers should view AVP-assisted procedures as experimental. Widespread adoption should not occur until Stage 2b trials confirm non-inferiority or superiority in patient-relevant outcomes.

The reasons for the limitations of this review are multifaceted. Having been launched as a consumer product for just over two years, the AVP is still in the early stages of medical application. Researchers must first address engineering challenges such as complex device connectivity, workflow integration, and ergonomic adaptation, which results in low reproducibility and hinders the rapid initiation of large-scale clinical trials. Second, the AVP has not yet obtained marketing approval from major regulatory bodies such as the FDA or NMPA as a diagnostic or surgical navigation device, meaning that conducting large-scale confirmatory studies currently faces multiple barriers including ethical review, medical liability determination, and informed consent. Consequently, most studies are published as technical demonstrations or feasibility reports rather than as rigorous clinical trials. At the same time, the rapid iteration of the AVP hardware means that researchers may encounter device updates before completing a moderately sized study, reducing data comparability and further discouraging investment in long-term, large-sample studies. Moreover, the ecosystem of specialized medical software for the AVP remains immature, lacking standardized surgical navigation modules, image fusion protocols, and objective evaluation metrics. Different research units adopt heterogeneous connectivity solutions and assessment tools, making it difficult to pool or cross-compare results.

Nevertheless, it must be recognized that the current lack of large-scale clinical data does not negate the application potential of the AVP, but rather represents a necessary phase in the journey of an emerging device toward evidence-based practice. To more objectively evaluate the true value of the AVP in clinical medicine and medical education, future efforts should establish standardized operating procedures, product performance metrics, and clinical outcome sets, conduct multicenter, prospective, randomized controlled trials or crossover design confirmatory trials, and encourage collaboration between device manufacturers and academic groups to initiate long-term registry studies once the hardware becomes relatively stable. Feasible strategies for integrating the AVP into existing medical device clinical trial regulatory pathways should also be explored. Relevant authorities need to clarify ethical and legal boundaries, standardize the attribution of responsibility for medical accidents caused by intraoperative device failure, ensure the authenticity of data records, and establish corresponding clinical use guidelines and liability insurance mechanisms, with the aim of promoting large-scale application of the AVP in clinical medicine and medical education.

## 8 Conclusions

Apple Vision Pro has shown preliminary application value in clinical medicine and medical education. Clinically, it has shown potential to support preoperative planning, intraoperative navigation, and postoperative rehabilitation through 3D visualization, real-time image fusion, and immersive interaction, with preliminary evidence of improved precision and efficiency. In education, it supports anatomy teaching, skills training, specialized practice, and remote collaboration, overcoming traditional resource constraints. However, its application faces multiple limitations, and most existing studies remain at IDEAL-D Stage 0–2a, with only two Stage 2b studies identified. High-level evidence for clinical benefit is still lacking. Future efforts should focus on hardware iteration, specialized software, multicenter RCTs, and standardization to transition AVP from technical demonstration to evidence-based clinical practice.

## Supplementary material

Supplementary Table S1 Relevant studies selected for this review. Among the studies applicable to the IDEAL-D framework, 4 case reports and 5 original research articles were published as Letters, Videos, Comments, Brief Reports, or Oral presentations. Based on their content, the type of study was classified as either “Case Report” or “Original Research”. For studies not applicable to the IDEAL-D framework, the “Number of Patients” and “Level of Evidence” columns were marked with a backslash (“\”).

## References

[1] Mastropasqua R Ruggeri ML Quarta A Tartaro R Vecchiarino L Corboli LV. New mixed reality headset: first exploratory use in intraocular surgery and telementoring. Ophthalmol Ther. (2025) 14(7):1597–609. 10.1007/s40123-025-01117-y 40214829 PMC12167422

[2] Olexa J Kim KT Saadon JR Rakovec M Evans M Cohen J. Apple vision pro augmented reality-assisted minimally invasive surgical treatment of spinal dural arteriovenous fistula. Cureus. (2024). 10.7759/cureus.63657 PMC11293944 39092384

[3] Waisberg E Ong J Masalkhi M Zaman N Sarker P Lee AG. The future of ophthalmology and vision science with the apple vision pro. Eye. (2024) 38(2):242–3. 10.1038/s41433-023-02688-5 37542175 PMC10810972

[4] Iwanaga J Loukas M Dumont AS Tubbs RS. A review of anatomy education during and after the COVID-19 pandemic: revisiting traditional and modern methods to achieve future innovation. Clinical Anatomy. (2021) 34(1):108–14. 10.1002/ca.23655 32681805 PMC7404762

[5] Gupta RK Pawa A. Beam me up, scotty! apple vision pro highlights how we could teleport ultrasound-guided regional anesthesia education into the future. Reg Anesth Pain Med. (2025) 50(4):379–80. 10.1136/rapm-2024-105424 38580337

[6] Sang AY Wang X Paxton L. Technological advancements in augmented, mixed, and virtual reality technologies for surgery: a systematic review. Cureus. (2024). 10.7759/cureus.76428 PMC11763273 39867005

[7] Nowak R Nowak M Rękas M Ali MJ. Intraoperative use of mixed reality (MR) in humans across surgical disciplines: a major review. 3D Print Med. (2025) 11(1):59. 10.1186/s41205-025-00305-7 41452379 PMC12746634

[8] Shanbhag NM Bin Sumaida A Al Shamisi K Balaraj K. Apple vision pro: a paradigm shift in medical technology. Cureus. (2024). 10.7759/cureus.69608 PMC11413724 39308843

[9] Marcus HJ Bennett A Chari A Day T Hirst A Hughes-Hallett A. IDEAL-D framework for device innovation: a consensus statement on the preclinical stage. Ann Surg. (2022) 275(1):73–9. 10.1097/SLA.0000000000004907 33856386 PMC8683254

[10] Jacob AM Böhner AMC Henkel A Aziz-Safaie T Oelmeier L Lenzen N. Optical see-through and video see-through head-mounted displays for percutaneous biopsies: a comparative phantom study. Cardiovasc Intervent Radiol. (2026) 49(5):970–9. 10.1007/s00270-026-04360-3 41946978 PMC13156115

[11] Bexten T Boehm A Miller J Yalamuri SM Read MD Simard T. Virtual ultrasound-guided central vascular access using apple vision pro: an evaluation and proof of concept. Cureus. (2025). 10.7759/cureus.87274 PMC12318436 40755649

[12] Miller J Bexten T Wiebe S Read M Simard T Yalamuri S. Augmented reality ultrasound-guided vascular access via apple vision pro. J Soc Cardiovasc Angiogr Interv. (2026) 5(8):105452. 10.1016/j.jscai.2026.105452 42626386 PMC13491326

[13] Tregobov N Jiang T Edgcumbe P Alhuwaymil A Yan JH Aludaini A. Augmented reality and ultrasonography-guided needle placement: feasibility and accuracy in a phantom model. J Diagn Med Sonogr. (2026) 42(4):293–7. 10.1177/87564793261418020

[14] Iribar-Zabala A Ruiz-Olalla-Del-Fresno J Rubio-Pérez I Moreta-Martínez R Beristain-Iraola A Pascau J. A comparative study of MR-guided needle insertion for surgical procedures: insights from HoloLens 2, magic leap 2 and apple vision pro. Healthc Technol Lett. (2026) 13(1):e70037. 10.1049/htl2.70037 41757261 PMC12934502

[15] Okachi S Ina T Horiguchi T Goto Y Hashimoto N Imaizumi K. Navigational bronchoscopy training with spatial computing: a simulation-based feasibility report using apple vision pro. Surg Innov. (2026):15533506261441953. 10.1177/15533506261441953 41964283

[16] Nagano S Momose K Kato T Ishii Y Yamaguchi S Hagi T. Immersive endoscopy: potential usefulness from an ergonomic perspective. Minim Invasive Ther Allied Technol. (2026):1–10. 10.1080/13645706.2026.2693303 42396688

[17] Kawaguchi H Yamasaki H. AR-guided probe positioning system for fNIRS using a head-mounted display. In: Ding J Fu L, editors. Neural Imaging and Sensing 2026. San Francisco, United States: SPIE (2026). p. 23. Available online at: https://www.spiedigitallibrary.org/conference-proceedings-of-spie/13835/3080193/AR-guided-probe-positioning-system-for-fNIRS-using-a-head/10.1117/12.3080193.full (Accessed September 13, 2026)

[18] Ma H Sun C Li Z Chen J Li R Wang Z. Augmented reality-assisted guide plate positioning in lumbar surgery using apple vision pro. In: 2025 IEEE Conference on Virtual Reality and 3D User Interfaces Abstracts and Workshops (VRW). Saint Malo, France: IEEE (2025). p. 1272–3. Available online at: https://ieeexplore.ieee.org/document/10972947/doi: 10.1109/VRW66409.2025.00282 (Accessed September 13, 2026).

[19] Javaheri H Fortes Rey V Lukowicz P Stavrou GA Karolus J Ghamarnejad O. Assessing the feasibility of using apple vision pro while performing medical precision tasks: controlled user study. JMIR XR Spatial Comput. (2025) 2:e73574. 10.2196/73574 PMC12671319 41340843

[20] Shen K Li W Xie Z. Preliminary application of apple vision pro (AVP) in laparoscopic gastrointestinal surgery. Int J Surg. (2025) 111(11):7898–905. 10.1097/JS9.0000000000003066 40679982 PMC12626454

[21] Shen KX Yang CB Wang C Ge XB Xing JP Sun BY. Exploration and practice of apple vision pro in medical education: application in laparoscopic surgery teaching. Chin J Dig Surg. (2025) 24(12):1566–71. 10.3760/cma.j.cn115610-20251011-00620

[22] Park DY Park SM Hashmi S Lee YP Bhatia N Oh M. Enhancing endoscopic spine surgery with intraoperative augmented reality: a case report. Int J Surg Case Rep. (2025) 131(C). 10.1016/j.ijscr.2025.111342 40279997 PMC12060460

[23] Hanks H-JE Kim MS Lin R Chen V Ton AT Mills E. Incorporating mixed reality head mounted display technology in biportal endoscopic lumbar surgery: an early feasibility study. Front Surg. (2026) 13:1772853. 10.3389/fsurg.2026.1772853 41869381 PMC12999923

[24] Bathini AR Dubey A Anastasi K Carlson B Parker JJ Fonseca R. Use of a mixed reality headset for full-endoscopic uniportal interlaminar resection of a lumbar facet cyst: 2-dimensional operative video. Oper Neurosurg. (2026). 10.1227/ons.0000000000001949 41705978

[25] Hanks H-J Chen V Choi C Horowitz J Hur J-W Mills E. Mixed reality in spine surgery: the apple vision pro in biportal endoscopic lumbar fusion. JoVE. (2026) 233:71067. 10.3791/71067 42546088

[26] Broderick RC Spurzem GJ Jeffery Reeves J Hollandsworth HM Sandler BJ Jacobsen GR. First use of augmented reality headset in minimally invasive general surgery: seeing is believing. Surg Endosc. (2025) 39(9):6055–60. 10.1007/s00464-025-11985-x 40640628 PMC12408766

[27] Liu Z Gu J Zhang Y Bai L Guan Y Ni B. Total laparoscopy-assisted total gastrectomy with Da Vinci robotic system conducted by robotic enhanced neurocomputing joint intelligence gastrointestinal surgery hub (RENJI-GISH): a preliminary clinical study and case report. World J Surg Onc. (2025) 23(1):86. 10.1186/s12957-025-03735-z PMC11907975 40087678

[28] Abu Zahra S Riaz A Kundu A Lewandowski RJ Gordon AC. Augmented reality-assisted cholangioscopy for biliary interventions: initial clinical experience in four cases. Surg Endosc. (2026) 40(8):7189–96. 10.1007/s00464-026-13023-w 42426392 PMC13457397

[29] Olexa J Trang A Cohen J Kim K Rakovec M Saadon J. The apple vision pro as a neurosurgical planning tool: a case report. Cureus. (2024). 10.7759/cureus.54205 PMC10942844 38496193

[30] Sekhon S Mitchell E Tran B Duchman B Spurzem G Broderick R. A24-03 augmented reality impact on perceived task load during bronchoscopy: a feasibility study. Am J Respir Crit Care Med. (2026) 212(Supplement_1):aamag162.3400. 10.1093/ajrccm/aamag162.3400

[31] Orione M Rubegni G Tartaro R Alberghina A Fallico M Orione C. Utilization of apple vision pro in ophthalmic surgery: a pilot study. Eur J Ophthalmol. (2025) 35(2):715–21. 10.1177/11206721241273574 39140319

[32] Korn TS Santiago C Fuller Y Said B. Apple vision pro as the intended primary intraoperative visualization platform for complete cataract surgery: a prospective single-center feasibility study. OPTH. (2026) 20:1–16. 10.2147/OPTH.S633394 PMC13429111 42544227

[33] El Jalbout ND Bansal R Shoji MK Cheng S D'Urso A Hosalkar HH. Apple vision pro as a wearable display for endoscopic dacryocystorhinostomy: surgical experience, efficiency, and cost. AJO International. (2026) 3(3):100288. 10.1016/j.ajoint.2026.100288

[34] Tsai YC Hsiao CC Chung-Wei Lin C Chern MC Huang SW. Apple vision pro-guided laparoscopic radio frequency ablation for liver tumors: the pioneer experience. Surg Innov. (2025) 32(3):312–4. 10.1177/15533506251316001 39853152

[35] Lan T Liu S Dai Y Luo J Han J Jin Y. Real-time image fusion and apple vision pro in laparoscopic microwave ablation of hepatic hemangiomas. npj Precis Onc. (2025) 9(1):79. 10.1038/s41698-025-00867-z PMC11926265 40113974

[36] Montoro Neto R Vicentini FC Ugino RTS Danilovic A Marchini GS Torricelli FCM. Mixed reality ultrasound-guided mini-ECIRS with apple vision Pro^TM^—first case report. Int Braz J Urol. (2025) 51(2):e20240610. 10.1590/s1677-5538.ibju.2024.0610 39913103 PMC11884634

[37] Wen CY Chi-Hsiang Wu J Tsai YC Huang SW. Apple vision pro used in retrograde intrarenal surgery: the pioneer experience in the real world. J Formos Med Assoc. (2026):S0929664626002895. 10.1016/j.jfma.2026.03.119 41934027

[38] Murphy PM An JY Wojdyla LM Searleman AC Khurana A Loehfelm TW. Diagnostic performance of a next-generation virtual/augmented reality headset: a pilot study of diverticulitis on CT. J Digit Imaging Inform Med. (2025) 38(5):3271–8. 10.1007/s10278-024-01292-7 PMC12572419 39495441

[39] Dandu C Creisher B Davis M Lin M Schenke S Aboian E. Integration of high-resolution mixed reality models into resident training. J Vasc Surg Cases Innov Tech. (2026) 12(2):102133. 10.1016/j.jvscit.2026.102133 41674916 PMC12887252

[40] Armstrong DG Bazikian S Armstrong AA Clerici G Casini A Pillai A. An augmented vision of our medical and surgical future, today? J Diabetes Sci Technol. (2024) 18(4):968–73. 10.1177/19322968241236458 38439541 PMC11307216

[41] Azar P Wong J Nikoo M Li VW Machado J To T. Utilizing apple vision pro for mindfulness intervention to reduce pain and anxiety in a hospitalized patient with mixed connective tissue disease: a case report. Cureus. (2026). 10.7759/cureus.107759 PMC13211975 42205645

[42] Radevic A Austerlitz J Gilbertson R Krishna S Colello M Johnson D. Novel intraoperative augmented reality-guided specimen localization in chest wall oncologic surgery: a case report. J Med Ext Real. (2026) 3:29941520261426873. 10.1177/29941520261426873 42558123 PMC13383645

[43] Chriqui S Ryan JF Luu BK Tran M Fergerson B You A. Patient perception of the use of the apple vision pro virtual reality headset in an anesthesiology informed consent process: prospective randomized controlled crossover trial. JMIR XR Spatial Comput. (2026) 3:e95806. 10.2196/95806 PMC13397004 42499682

[44] Zahra SA Antalek M Kundu A Nadeem A Schenke S Finos K. Augmented and virtual reality for preprocedural planning in hepatic transarterial interventional oncology: a pilot study in 50 cases. J Vasc Interv Radiol. (2026) 37(11):108979. 10.1016/j.jvir.2026.108979 42492769

[45] Xu B Wu W. Co-designing a multimodal AR assistant for interventional radiology workflows. In: Proceedings of the Extended Abstracts of the 2026 CHI Conference on Human Factors in Computing Systems. Barcelona, Spain: ACM (2026). p. 1–5. Available online at: https://dl.acm.org/doi/10.1145/3772363.3799005 (Accessed September 13, 2026).

[46] Dhawan R Bikmal A Shay D. From virtual to reality: apple vision pro’s applications in plastic surgery. J Plast Reconstr Aesthet Surg. (2024) 94:43–5. 10.1016/j.bjps.2024.04.068 38749368

[47] Masalkhi M Waisberg E Ong J Zaman N Sarker P Lee AG. Apple vision pro for ophthalmology and medicine. Ann Biomed Eng. (2023) 51(12):2643–6. 10.1007/s10439-023-03283-1 37332003

[48] Cheng W Lynn MH Pundlik S Almeida C Luo G Houston K. A smartphone ocular alignment measurement app in school screening for strabismus. BMC Ophthalmol. (2021) 21(1):150. 10.1186/s12886-021-01902-w 33765984 PMC7992982

[49] Masalkhi M Ong J Waisberg E Dervan E Lee AG. Apple vision pro’s new technology as a head-mounted perimetry device for glaucoma and other potential applications. Pan-Am J Ophthalmol. (2024) 6(3). 10.4103/pajo.pajo_32_24

[50] Guirguis CA Tung JK. Apple vision pro: potential applications of mixed reality in dermatology. Int J Dermatol. (2024) 63(11). 10.1111/ijd.17437 39123281

[51] Kaye AD Islam RK Islam KN Khachemoune A Haas C Barrie S. Apple vision pro and its implications in mohs micrographic surgery: a narrative review. Cureus. (2024). 10.7759/cureus.71440 PMC11560411 39544562

[52] Pinilla A Garcia J Raffe W Voigt-Antons JN Spang RP Möller S. Affective visualization in virtual reality: an integrative review. Front Virtual Real. (2021) 2:630731. 10.3389/frvir.2021.630731

[53] Zhang Z Giménez Mateu LG Fort JM. Apple vision pro: a new horizon in psychological research and therapy. Front Psychol. (2023) 14:1280213. 10.3389/fpsyg.2023.1280213 38023044 PMC10651722

[54] Department of Orthopaedics, ACS Medical College and Hospital, Dr. MGR Educational and Research Institute, Chennai, Tamil Nadu, India, Jeyaraman M, Jeyaraman N, Department of Orthopaedics, ACS Medical College and Hospital, Dr. MGR Educational and Research Institute, Chennai, Tamil Nadu, India, Ramasubramanian S, Department of Orthopaedics, Government Medical College, Omandurar Government Estate, Chennai, Tamil Nadu, India. Enhancing orthopedic rehabilitation: the emergence and impact of virtual reality technology. J Orthop Case Rep. (2024) 14(4):1–6. 10.13107/jocr.2024.v14.i04.4338 PMC11043980 38681925

[55] Diriba Kenea C Gemechu Abessa T Lamba D Bonnechère B. Technological features of immersive virtual reality systems for upper limb stroke rehabilitation: a systematic review. Sensors. (2024) 24(11):3546. 10.3390/s24113546 38894337 PMC11175221

[56] Paladugu P Kumar R Ong J Waisberg E Sporn K. Virtual reality-enhanced rehabilitation for improving musculoskeletal function and recovery after trauma. J Orthop Surg Res. (2025) 20(1):404. 10.1186/s13018-025-05705-3 40269873 PMC12016257

[57] Alfaya FF Reddy RS Alkhamis BA Kandakurti PK Mukherjee D. Shoulder proprioception and its correlation with pain intensity and functional disability in individuals with subacromial impingement syndrome—a cross-sectional study. Diagnostics. (2023) 13(12):2099. 10.3390/diagnostics13122099 37370994 PMC10296834

[58] Álvarez De La Campa Crespo M Donegan T Amestoy-Alonso B Just A Combalía A Sanchez-Vives MV. Virtual embodiment for improving range of motion in patients with movement-related shoulder pain: an experimental study. J Orthop Surg Res. (2023) 18(1):729. 10.1186/s13018-023-04158-w PMC10523655 37752613

[59] Kouijzer MMTE Kip H Bouman YHA Kelders SM. Implementation of virtual reality in healthcare: a scoping review on the implementation process of virtual reality in various healthcare settings. Implement Sci Commun. (2023) 4(1):67. 10.1186/s43058-023-00442-2 37328858 PMC10276472

[60] Veerla JP Guttikonda PS Shang HH Nasr MS Torres C Luber JM. Beyond the monitor: mixed reality visualization and multimodal AI for enhanced digital pathology workflow. In: 2026 IEEE 23rd International Symposium on Biomedical Imaging (ISBI). London, United Kingdom: IEEE (2026). p. 1–5. Available online at: https://ieeexplore.ieee.org/document/11515391/doi: 10.1109/ISBI61048.2026.11515391 (Accessed September 13, 2026).

[61] Waisberg E Ong J Masalkhi M Zaman N Sarker P Lee AG. Apple vision pro and the advancement of medical education with extended reality. Can Med Ed J. (2023). 10.36834/cmej.77634 PMC10961128 38528897

[62] Patil S Lalwani V Mahmud BU Carr SM Woodcock J Grellinger K. A mixed-reality approach to cardiovascular anatomy education. Future Internet. (2026) 18(6):314. 10.3390/fi18060314

[63] McGaghie WC Issenberg SB Petrusa ER Scalese RJ. A critical review of simulation-based medical education research: 2003–2009. Med Educ. (2010) 44(1):50–63. 10.1111/j.1365-2923.2009.03547.x 20078756

[64] Waisberg E Ong J Masalkhi M Zaman N Sarker P Lee AG. Apple vision pro and why extended reality will revolutionize the future of medicine. Ir J Med Sci. (2024) 193(1):531–2. 10.1007/s11845-023-03437-z 37365445

[65] Isshiki M Yamanishi J Kinoshita K Mitani S. Development and evaluation of a stepwise learning support system for surgical education using augmented reality. IEEJ Trans EIS. (2026) 146(7):664–71. 10.1541/ieejeiss.146.664

[66] Jayawardene W Kumbalatara C Kase M Goodson A Park A Willett J. Development and testing of a self-paced virtual reality-based naloxone training. Subst Use Misuse. (2026) 61:471–6. 10.1080/10826084.2025.2560073 40960822

[67] Jayawardene W Magnuson R Kumbalatara C Kase M Park A Goodson A. Usability of mixed reality for naloxone training: iterative development and field testing of ReviveXR. Healthcare. (2025) 13(12):1449. 10.3390/healthcare13121449 40565476 PMC12192816

[68] Waisberg E Ong J Masalkhi M Zaman N Sarker P Lee AG. Apple vision pro: the future of surgery with advances in virtual and augmented reality. Ir J Med Sci. (2024) 193(1):345–6. 10.1007/s11845-023-03457-9 37422552

[69] Sporn K Kumar R Paladugu P Ong J Sekhar T Vaja S. Artificial intelligence in orthopedic medical education: a comprehensive review of emerging technologies and their applications. IME. (2025) 4(2):14. 10.3390/ime4020014

[70] Stauffer T Gutiez B Babst R Lohmeyer Q Meboldt M. Augmented reality enhanced physical simulation for femoral nailing training. Int J CARS. (2026) 21:1533–40. 10.1007/s11548-026-03646-8 PMC13694326 42250193

[71] Mohamed AA Lucke-Wold B. Apple intelligence in neurosurgery. Neurosurg Rev. (2024) 47(1):327. 10.1007/s10143-024-02568-w 39004685

[72] Zhou AE Klufas T Feng H. Leveraging apple vision pro for dermatologic surgery education and intraoperative recording. Indian Dermatol Online J. (2025) 17:294–5. 10.4103/idoj.idoj_692_25 41403068 PMC13021181

[73] Erdem O Gürel MS. A practical and affordable surgical light-integrated camera system for dermatologic surgery video recordings. J Am Acad Dermatol. (2025) 92(2):e39–40. 10.1016/j.jaad.2024.08.065 39303922

[74] Kantor J. Use of 3-dimensional 360-degree virtual reality video in dermatologic and reconstructive surgery and mohs micrographic surgery. J Am Acad Dermatol. (2021) 85(2):e67–8. 10.1016/j.jaad.2017.05.044 34006437

[75] Ahuja A Yang S Paredes Iii A Tu D. Virtual reality in ophthalmic surgical education: current innovations and future perspectives. OPTH. (2026) 20:1–8. 10.2147/OPTH.S593471 PMC13221436 42221038

[76] Amirpour H Putzgruber-Adamitsch D Schoeffmann K. Depth-enabled inspection of medical videos. In: Proceedings of the 33rd ACM International Conference on Multimedia. Dublin Ireland: ACM (2025). p. 13504–6. Available online at: https://dl.acm.org/doi/10.1145/3746027.3754469 (Accessed September 13, 2026).

[77] Tomiuk E Miró J Duong L. Evaluation of eye-tracking capabilities in apple vision pro for training in hybrid ventricular septal defect procedures. In: Rettmann ME Siewerdsen JH, editors. Medical Imaging 2025: Image-Guided Procedures, Robotic Interventions, and Modeling. San Diego, United States: SPIE (2025). p. 18. Available online at: https://www.spiedigitallibrary.org/conference-proceedings-of-spie/13408/3047251/Evaluation-of-eye-tracking-capabilities-in-Apple-Vision-Pro-for/10.1117/12.3047251.full (Accessed September 13, 2026).

[78] Luijten G Faray de Paiva L Krueger S Brost A Mazilescu L Santos AS. From screen to space: evaluating siemens’ cinematic reality application for medical imaging on the apple vision pro. Front Virtual Real. (2026) 6:1666614. 10.3389/frvir.2025.1666614

[79] Li C. Apple vision pro as an augmentative tool in surgery: a double-edged scalpel. Ann Med Surg. (2024) 86(11):6407–8. 10.1097/MS9.0000000000002645 PMC11543186 39525735

[80] Goh LC Ong HY Abu Bakar MZ. 5G-enabled XR live broadcasting of transoral robotic surgery (TORS): a feasibility study of an indoor small-cell architecture. J Robotic Surg. (2026) 20(1):238. 10.1007/s11701-026-03191-1 PMC12891050 41667903

[81] Nowak R Nowak-Gospodarowicz I Rękas M Ali MJ. Virtual reality and mixed reality-assisted endoscopic DCR in extremely complex lacrimal obstructions. Laryngoscope. (2024) 134(8):3508–15. 10.1002/lary.31399 38511475

[82] Huang H Yin J Lv F Lin Y Zou J. A study on the impact of open source metaverse immersive teaching method on emergency skills training for medical undergraduate students. BMC Med Educ. (2024) 24(1):859. 10.1186/s12909-024-05862-9 PMC11316355 39123134

[83] Edgcumbe PM Jiang T Ho JF Martin ML Stein MJ Isaac KV. Intraoperative surgical guidance for DIEP flap harvest using augmented reality. Plast Reconstr Surg. (2025) 155(6):932–5. 10.1097/PRS.0000000000011863 39661028 PMC12105944

[84] Egger J Gsaxner C Luijten G Chen J Chen X Bian J. Is the apple vision pro the ultimate display? A first perspective and survey on entering the wonderland of precision medicine. JMIR Serious Games. (2024) 12:e52785. 10.2196/52785 39292499 PMC11447423

[85] Sadowski J Wolaniuk S Klaudel T Sikorski M Wąsik M. A review of the latest information on the implementation of virtual reality for medical applications including: educational, intraoperative, diagnostic, rehabilitation and therapeutic applications with a consideration of selected reports on apple vision pro. Health Technol. (2025) 15(1):29–40. 10.1007/s12553-024-00935-7

[86] Volin J Viswanathan V Krupinski EA Safdar N Silverman R Narayan V. Feasibility of using the apple vision pro for diagnostic radiology: user experience and perceived clinical utility. J Digit Imaging Inform med. (2025). 10.1007/s10278-025-01745-7 PMC13694668 41249669

