# Supplementary material for: Early applications of apple vision pro in clinical medicine and medical education: a scoping review
Source: Front Med (Lausanne). 2026 Oct 16;13:1909038. doi: 10.3389/fmed.2026.1909038 (PMC13721002; doi:10.3389/fmed.2026.1909038)
Supplement: Supplementary file 1 [file Table1.docx]

| Author(s) | Country | Year | Type of Study | Topic | Number of Patients | Level of Evidence | Main limitations |
| --- | --- | --- | --- | --- | --- | --- | --- |
| Jacob et al. (10) | Germany | 2026 | Original Research | A comparison was made between optical see‑through (Magic Leap 2) and video see‑through (AVP) head‑mounted displays regarding their adjunctive value for CT‑guided percutaneous biopsy in an abdominal phantom. | 0 | Stage 0 | The study was conducted in a static abdominal phantom, did not account for patient motion such as respiration, and used a limited number of punctures per condition. |
| Bexten et al. (11) | USA | 2025 | Original Research | Evaluation and proof of concept of virtual ultrasound-guided central vascular access using AVP. | 0 | Stage 0 | The study was conducted on a static phantom and did not account for clinical factors such as tissue deformation and respiratory motion in real patients. |
| Miller et al. (12) | USA | 2026 | Original Research | User experience of AVP‑guided ultrasound vascular access assessed in 21 physicians on phantoms and live animals. | 0 | Stage 0 | This study was limited by its single‑arm design, small sample size, absence of objective performance metrics, and exclusive reliance on subjective user feedback. |
| Tregobov et al. (13) | Canada | 2026 | Original Research | AVP-based augmented reality ultrasound guidance enabled accurate and feasible needle placement in a phantom, comparable to conventional sonography. | 0 | Stage 0 | The main limitations are the small sample, phantom-based design, ceiling effect, and issues with visualization, comfort, and technical reliability. |
| Iribar-Zabala et al. (14) | Spain | 2026 | Original Research | In a phantom needle insertion navigation task, the Magic Leap 2 achieved the highest success rate and best usability, while the AVP was unsuitable for navigation due to unstable tracking and poor comfort. | 0 | Stage 0 | Phantom-based study, small sample size, and not validated in a real clinical setting. |
| Okachi et al. (15) | Japan | 2026 | Letter | This study validates the technical feasibility of using AVP for navigational bronchoscopy simulation training. | 0 | Stage 0 | Only two experienced operators were involved, with no novice trainees, and the actual training effectiveness was not evaluated. |
| Nagano et al. (16) | Japan | 2026 | Original Research | AVP immersive endoscopy in 11 endoscopists: reduced procedure time, mental workload, and neck/shoulder muscle activation versus non-ergonomic setting; outperformed ergonomic setting in certain postural muscles. | 0 | Stage 0 | Small sample size, ex vivo model with short task duration, lack of objective latency/image quality metrics, and need for clinical validation. |
| Kawaguchi et al. (17) | Japan | 2026 | Conference Paper | This study used AVP for AR-guided fNIRS probe positioning and validated its accuracy on a head mannequin. | 0 | Stage 0 | The main limitation is that accuracy was validated only on a head mannequin, not in real subjects or clinical settings, and the CG position shifted slightly depending on viewing angle. |
| Ma et al. (18) | China | 2025 | Conference Paper | An AVP-based augmented reality system enables accurate guide plate positioning in lumbar surgery. | 0 | Stage 0 | Tthe study was conducted only on 3D-printed phantoms, ex vivo porcine specimens, and two live pigs, with a small sample size, no human clinical validation, and reliance on manual fine-tuning and fiducial marker tracking. |
| Javaheri et al. (19) | Germany | 2025 | Original Research | The AVP significantly increased task completion time and cognitive load during phantom suturing compared to HoloLens 2 and no headset, yet without compromising suturing quality. | 0 | Stage 0 | The study was performed on a suture phantom rather than real patients, excluded participants who required corrective eyewear due to AVP’s design, and did not assess any digital overlay applications. |
| Park et al. (22) | USA | 2025 | Case Report | A patient with lumbar spinal stenosis successfully underwent AVP‑assisted biportal endoscopic spine surgery. | 1 | Stage 1 | Single case report without a control group, limiting generalizability. |
| Hanks et al. (23) | USA | 2026 | Original Research | In 40 cases of biportal endoscopic lumbar surgery, the AVP did not increase surgeon cognitive load and showed favorable clinical outcomes. | 40 | Stage 2a | No control group, single-surgeon experience, and only 3 months of follow-up. |
| Bathini et al. (24) | USA | 2026 | Case Report | A patient with a lumbar facet cyst successfully underwent AVP‑assisted full‑endoscopic resection. | 1 | Stage 1 | Single case report without a control group, limiting generalizability. |
| Hanks et al. (25) | USA | 2026 | Case Report | AVP-assisted biportal endoscopic TLIF for L4-5 spondylolisthesis with favorable short-term outcomes. | 1 | Stage 1 | Single case report without a control group, limiting generalizability. |
| Broderick et al. (26) | USA | 2025 | Original Research | In 41 minimally invasive surgery cases, using the AVP as the primary monitor was feasible, with low cognitive load and no complications. | 41 | Stage 2a | Single-center, small sample, and no control group. |
| Shen et al. (20) | China | 2025 | Original Research | In 22 laparoscopic colorectal cancer surgeries assisted by AVP, all procedures were successful, with a mean operative time of 189 minutes, no complications, and SUS scores ranging from 90 to 100. | 22 | Stage 2a | Single-center, no control group, and limited sample size. |
| Liu et al. (27) | China | 2025 | Case Report | Total laparoscopy-assisted total gastrectomy utilizing Da Vinci robotic system. | 1 | Stage 1 | Single case report without a control group, limiting generalizability. |
| Zahra et al. (28) | USA | 2026 | Case Report | AVP-assisted cholangioscopy for biliary interventions in four cases. | 4 | Stage 1 | Single case report without a control group, limiting generalizability. |
| Olexa et al. (2) | USA | 2024 | Case Report | Augmented reality-assisted minimally invasive surgical treatment of spinal dural arteriovenous fistula using AVP. | 1 | Stage 1 | Single case report without a control group, limiting generalizability. |
| Olexa et al. (29) | USA | 2024 | Case Report | The use of AVP as a neurosurgical planning tool. | 1 | Stage 1 | Single case report without a control group, limiting generalizability. |
| Sekhon et al. (30) | Japan | 2026 | Conference Paper | This feasibility study evaluated the impact of augmented reality using AVP on perceived task load during bronchoscopy in 30 patients. | 30 | Stage 2a | its small sample size (30 patients), single-center feasibility design, absence of a control group, and reliance on subjective workload measures, requiring confirmation in larger randomized trials. |
| Orione et al. (31) | Italy | 2024 | Original Research | In 10 eyelid surgeries assisted by AVP, high usability scores were achieved, but visual comfort and information clarity were low. | 10 | Stage 2a | Small sample size and no validated dedicated questionnaire. |
| Mastropasqua et al. (1) | Italy | 2025 | Original Research | In 9 intraocular surgeries, AVP-assisted procedures were successful, and the feasibility of remote guidance was validated. | 9 | Stage 1 | Small sample size, predominantly subjective assessment, and lack of a control group. |
| Korn et al. (32) | USA | 2026 | Original Research | AVP as the primary visualization platform for cataract surgery in 45 patients with favorable feasibility outcomes. | 45 | Stage 2a | Single-center, single-arm design without a control group, and no objective latency or ergonomic measurements. |
| El Jalbout et al. (33) | USA | 2026 | Original Research | This study evaluated the AVP as a wearable intraoperative display for endoscopic dacryocystorhinostomy, comparing surgical efficiency, surgeon workload, and cost with conventional monitor-based endoscopy. | 32 | Stage 2a | Single-center, single-arm design without a control group, and no objective latency or ergonomic measurements. |
| Tsai et al. (34) | China | 2025 | Case Report | In a patient with liver cancer, AVP-assisted laparoscopic ultrasound-guided radiofrequency ablation was performed, with a probe localization time of 41 seconds, and the surgery was successful. | 1 | Stage 1 | Single case report without a control group, limiting generalizability. |
| Lan et al. (35) | China | 2025 | Original Research | An AVP real‑time image fusion navigation system was developed, increasing the phantom puncture success rate from 42.5% to 90%; all eight patients with hepatic hemangiomas underwent successful ablation. | 8 | Stage 1 | Small sample size, no long-term follow-up, and the phantom experiment cannot fully simulate real respiratory motion and tissue deformation. |
| Montoro Neto et al. (36) | Brasil | 2025 | Case Report | A patient with kidney stones successfully underwent AVP‑assisted ultrasound‑guided Mini‑ECIRS surgery. | 1 | Stage 1 | Single case report without a control group, limiting generalizability. |
| Wen et al. (37) | China | 2026 | Case Report | A patient with kidney stones successfully underwent AVP‑assisted retrograde intrarenal surgery (RIRS). | 1 | Stage 1 | Single case report without a control group, limiting generalizability. |
| Murphy et al. (38) | USA | 2025 | Original Research | The diagnostic accuracy of the AVP headset for CT diverticulitis was comparable to that of the desktop (AUC 0.93 vs. 0.94), but interpretation time was longer (median 57 s vs. 31 s). | 110 | Stage 2b | The headset was not calibrated to the DICOM grayscale standard, the reading order was fixed (headset first), and there was no pathological gold standard. |
| Dandu et al. (39) | USA | 2026 | Case Report | This study demonstrates the use of AVP combined with 3D reconstruction models to enhance vascular surgery residents' spatial understanding of complex anatomy. | 1 | Stage 1 | Single case report without a control group, limiting generalizability. |
| Armstrong et al. (40) | USA | 2024 | Case Report | In two limb salvage procedures, the AVP assisted intraoperative decision-making, team communication, and teaching, but its real-time pass-through resolution was insufficient to support highly dexterous manipulation. | 2 | Stage 1 | Single case report without a control group, limiting generalizability. |
| Azar et al. (41) | Canada | 2026 | Case Report | AVP‑delivered mindfulness intervention for pain and anxiety in a patient with mixed connective tissue disease. | 1 | Stage 1 | Single case report without a control group, limiting generalizability. |
| Radevic et al. (42) | USA | 2026 | Case Report | AVP‑guided holographic reorientation of a specimen‑derived 3D model into the resection bed during staged chest wall reconstruction in a patient with sarcoma. | 1 | Stage 1 | Single case report without a control group, limiting generalizability. |
| Chriqui et al. (43) | USA | 2026 | Original Research | AVP during preoperative informed consent in 60 patients: reduced SCQ scores and eye contact satisfaction versus no headset. | 60 | Stage 2b | Single‑center study, predominantly White and older cohort, brief preprocedural encounters, and potential carryover effects without a washout period. |
| Zahra et al. (44) | USA | 2026 | Original Research | AVP‑based AR/VR preprocedural planning in 50 transarterial hepatic oncology cases: 5‑min median planning time and 46% change in procedural approach. | \ | \ | Single‑center design, subjective outcomes, and only 3 participating operators, without objective metrics or comparative data. |
| Xu et al. (45) | USA | 2026 | Conference Paper | This paper presents PRISM, an AVP-based multimodal AR assistant co-designed with interventional radiologists to support sterile, in-procedure imaging interaction in interventional radiology workflows. | \ | \ | Only theoretical discussion, lacking clinical data validation. |
| Dhawan et al. (46) | USA | 2024 | Correspondence | Discusses the potential applications of AVP in plastic surgery, including surgical simulation, preoperative planning, intraoperative decision support, and patient communication. | \ | \ | Only theoretical discussion, lacking clinical data validation. |
| Waisberg et al. (3) | Ireland | 2023 | Comment | Explores the potential applications of AVP in ophthalmic screening, low vision assistance, strabismus treatment, and astronaut eye health monitoring. | \ | \ | Only theoretical discussion, lacking clinical data validation. |
| Masalkhi et al. (47) | Ireland | 2025 | Letter | Explores the potential applications of AVP in ophthalmic vision screening, low vision assistance, strabismus rehabilitation, and neurovestibular rehabilitation. | \ | \ | Only theoretical discussion, lacking clinical data validation. |
| Masalkhi et al. (49) | Ireland | 2024 | Review | Apple Vision Pro’s new technology as a head‐mounted perimetry device for glaucoma and other potential applications. | \ | \ | Insufficient clinical validation, unresolved privacy and security issues, and unverified compatibility for perimetry use. |
| Guirguis et al. (50) | USA | 2024 | Correspondence | Exploring the potential applications of AVP in dermatology, including Mohs surgery, cosmetic injections, and psoriasis quantification. | \ | \ | Only theoretical discussion, lacking clinical data validation. |
| Kaye et al. (51) | USA | 2024 | Review | Exploring the potential applications of AVP in preoperative planning, intraoperative guidance, and postoperative analysis in Mohs micrographic surgery. | \ | \ | Narrative review, lacking original clinical data validation. |
| Zhang et al. (53) | Spain | 2023 | Opinion | Exploring the potential applications of AVP in psychological research and telepsychotherapy. | \ | \ | Only theoretical discussion, lacking experimental or clinical validation data. |
| Paladugu et al. (56) | USA | 2025 | Review | Exploring the potential applications of virtual reality technologies such as AVP in orthopedic rehabilitation for diagnosis, personalized treatment, and real-time feedback. | \ | \ | Narrative review, lacking original clinical data validation. |
| Veerla et al. (60) | USA | 2026 | Conference Paper | This paper presents PathVis, a mixed-reality platform for AVP that integrates immersive whole-slide image visualization with multimodal AI agents to streamline digital pathology workflows. | \ | \ | Only theoretical discussion, lacking clinical data validation. |
| Waisberg et al. (61) | UK | 2024 | Comment | AVP has the potential to revolutionize medical education through VR/AR technology, including clinical simulation, virtual anatomy, and visual experience of diseases. | \ | \ | Only theoretical discussion, lacking clinical data validation. |
| Patil et al. (62) | USA | 2026 | Original Research | This paper presents a reproducible mixed-reality anatomy learning platform on AVP that integrates the open-source Z-Anatomy atlas with a context-grounded RAG voice assistant bounded by Terminologia Anatomica, using cardiovascular anatomy as the case domain. | \ | \ | Only theoretical discussion, lacking clinical data validation. |
| Waisberg et al. (64) | Ireland | 2023 | Letter | Outlines the revolutionary potential of extended reality technologies such as AVP in medical education, surgical training, telemedicine, and rehabilitation. | \ | \ | Only theoretical discussion, lacking clinical data validation. |
| Gupta et al. (5) | USA | 2025 | Letter | AVP is expected to revolutionize ultrasound-guided regional anesthesia education through immersive AR technology. | \ | \ | Only theoretical discussion, lacking clinical data validation. |
| Isshiki et al. (65) | Japan | 2026 | Original Research | This study developed and evaluated a stepwise learning support system using augmented reality for surgical education. | \ | \ | Only theoretical discussion, lacking clinical data validation. |
| Jayawardene et al. (66) | USA | 2025 | Original Research | Developed and tested a self-paced mixed reality naloxone training system (VeNT) based on the AVP, which significantly improved participants' knowledge, confidence, and willingness to intervene. | \ | \ | Single-group pre-post design, lack of a control group, limited sample size, and no assessment of long-term effects. |
| Jayawardene et al. (67) | USA | 2025 | Original Research | Developed and tested an AVP-based mixed reality naloxone training system, ReviveXR, which significantly improved participants' knowledge, confidence, and willingness to intervene. | \ | \ | Single-group pre-post design, lack of a control group, limited sample size, and no assessment of long-term effects. |
| Waisberg et al. (68) | Ireland | 2023 | Letter | Explores the potential of AVP to enable real‑time data access, simulation training, and objective competency assessment in surgery. | \ | \ | Only theoretical discussion, lacking clinical data validation. |
| Shen et al. (21) | China | 2025 | Original Research | This study introduced the technical optimization of AVP in laparoscopic surgery teaching and its effects on improving trainees' skills and examination scores. | \ | \ | Single-center, non-randomized controlled design, limited sample size, and lack of long-term skill retention assessment. |
| Sporn et al. (69) | USA | 2025 | Review | A Comprehensive Review of Artificial Intelligence and Emerging Technologies in Orthopedic Medical Education. | \ | \ | The current evidence remains preliminary, heterogeneous, and constrained by small samples, short follow-up, high costs, and unresolved ethical and regulatory issues. |
| Stauffer et al. (70) | Switzerland | 2026 | Original Research | AR‑enhanced physical simulator for femoral nailing training using AVP, with significant learning effects in task efficiency and fluoroscopy use, and experts outperforming non‑experts. | \ | \ | Only theoretical discussion, lacking clinical data validation. |
| Mohamed et al. (71) | USA | 2024 | Correspondence | Exploring the potential applications of Apple Intelligence in neurosurgical education, patient care, and work efficiency. | \ | \ | Only theoretical discussion, lacking clinical data validation. |
| Zhou et al. (72) | USA | 2025 | Letter | AVP can be used in dermatologic surgery education, providing hands-free, first-person high-quality intraoperative video recording. | \ | \ | Only theoretical discussion, lacking clinical data validation. |
| Ahuja et al. (75) | USA | 2026 | Original Research | Perspective review of VR/AR in ophthalmic surgical education, comparing established and emerging platforms, and identifying validation gaps. | \ | \ | Only theoretical discussion, lacking clinical data validation. |
| Amirpour et al. (76) | Ireland | 2025 | Conference Paper | This study evaluated stereoscopic cataract surgery videos viewed through AVP and Meta Quest 3, showing improved depth perception and spatial awareness for ophthalmic surgical training. | \ | \ | Only theoretical discussion, lacking clinical data validation. |
| Tomiuk et al. (77) | Canada | 2025 | Conference Paper | This study quantitatively evaluated the eye-tracking accuracy and precision of AVP for training in hybrid ventricular septal defect procedures. | \ | \ | Only theoretical discussion, lacking clinical data validation. |
| Luijten et al. (78) | Germany | 2025 | Original Research | This study evaluated the usability and clinical integration potential of Siemens’ Cinematic Reality application on the Apple Vision Pro for immersive medical imaging visualization. | \ | \ | Only theoretical discussion, lacking clinical data validation. |
| Cheng Li. (79) | China | 2024 | Letter | As a surgical assistive tool, AVP offers advantages such as enhanced visualization and remote collaboration, but faces challenges including cost, blind spots, battery life, and comfort. | \ | \ | Only theoretical discussion, lacking clinical data validation. |
| Goh et al. (80) | Malaysia | 2025 | Original Research | This study validated that an indoor 5G small‑cell architecture enables low‑latency, XR headset‑supported live broadcasting of transoral robotic surgery. | \ | \ | Single‑session, single‑case broadcast; no independent end‑to‑end latency measurement, and lack of objective assessment of learning outcomes. |
| Egger et al. (84) | Germany | 2024 | Original Research | Evaluated the potential and limitations of AVP in precision medicine through expert surveys and hardware comparisons. | \ | \ | Small sample size, only a preliminary experience report, lacking large‑scale clinical validation. |
| Sadowsk et al. (85) | Poland | 2025 | Review | This review summarizes recent applications of virtual reality in medical education, intraoperative, diagnostic, rehabilitation, and therapeutic settings, with selected consideration of Apple Vision Pro. | \ | \ | The main limitations of this review are its non-systematic design, heterogeneous and mostly small-scale short-term studies, and the lack of high-quality randomized controlled evidence, especially for Apple Vision Pro, whose clinical application remains in its early stages. |
| Volin et al. (86) | USA | 2025 | Original Research | Evaluated the feasibility, user experience, and perceived clinical value of using AVP for medical image viewing among radiology residents. | \ | \ | Small sample size, single-center design, predominantly residents, lack of objective diagnostic performance metrics and long-term usage assessment. |
| Shanbhag et al. (8) | ARE | 2024 | Review | This article reviews the potential applications of AVP in medical fields including ophthalmology, psychotherapy, medical education, and neurosurgery. | \ | \ | The review is based on concepts and early reports, lacking empirical data and clinical validation. |
